# Virus-related Knowledge in Covid-19 Times - Results from two Cross-sectional Studies in Austria and Implications for School

**DOI:** 10.7150/ijbs.69224

**Published:** 2022-01-31

**Authors:** Marc Bracko, Uwe Karsten Simon

**Affiliations:** Centre for Biology Teacher Education, Karl-Franzens-University Graz, Schubertstr. 51a, 8010 Graz, Austria.

**Keywords:** Covid-19, virology, vaccination, knowledge, school, public health

## Abstract

Viruses have become a prominent issue in public health, politics and economics due to the Covid-19 pandemic. Yet they have still met little attention in educational research, although misconceptions concerning viruses may contribute to antibiotics misuse, disbelief in existence of viruses and distrust in vaccination. We investigated knowledge and attitudes in Austria concerning Covid-19, viruses in general and vaccination. We conducted two cross-sectional online surveys. Study A was performed Austrian-wide (N = 1027), study B specifically targeted Austrian students from middle and high schools (N = 1728). Several participants did not believe in the existence of SARS-CoV-2. General vaccination damage was highly overrated. Many defined viruses as unicellular organisms or bacteria, and 6-10 % believed that they can be killed by antibiotics. Very many participants were unable to identify, whether a specific disease was caused by a virus or another pathogen. Knowledge was significantly correlated with level of education/grade and interest in virology. Additionally, willingness to become vaccinated was significantly correlated with knowledge. Many participants felt insufficiently informed about viruses at school. We strongly recommend that virus-related school education must highly improve to enable the population to correctly assess health-related information, counter fake news and come to scientifically informed decisions.

## Introduction

Information about SARS-CoV-2, the causal agent of Covid-19, has been flooding the public almost continuously for the past two years, and public, political and scientific interest in virology has surged to a peak probably unreached since the HIV-outbreak in the early 1980s. Thus, one may assume that knowledge about Covid-19, but also concerning virology in general is widespread, detailed and well-grounded. And yet, does the public understand viruses any better than before the outbreak of Covid-19? Has the pandemic led to a thorough understanding of viruses at school?

These questions are not academic. A basic understanding of virology is essential for many health-related decisions. For example, if the presence of a particular virus is not believed in, why should certain measures preventing its spread be respected? Even though they may not represent the majority, there are people south and north of the equator who do not accept the danger or even existence of the SARS-CoV-2 virus 1, 2, 3, 4, 5. But even those who do may be uncertain about how to deal with it. The World Health Organisation (WHO) has collected a number of Corona myths: https://www.who.int/emergencies/diseases/novel-coronavirus-2019/advice-for-public/myth-busters 6. One recurrent topic is the use of antibiotics against this pathogen. This is no outlier. The few studies available analysing virus-related knowledge in depth show that many people have severe difficulties in distinguishing (non-living) viruses from (living) bacteria 7, 8. Thus, it is not surprising that almost half of the interviewed Europeans in a study conducted by the European Commission believed that cold and flu, both caused by viruses, can be treated with antibiotics 7, with similar figures reported from Georgia 9, South Korea 10 or New Zealand 11. Even about 20% of participants of a study among Italian medical students thought that viral infections may be treated with antibiotics 12.

Along this line, many confound viral and bacterial diseases, define viruses as living micro-organisms, and have severe misconceptions as to their replication and spread 8. Yet mistakenly taking antibiotics when suffering from a virus infection is by no means harmless. Is has been calculated that about 700, 000 people die from antibiotics-resistant bacterial strains worldwide 13, with more than 35, 000 annual deaths in the U.S. 14 and 33, 000 annual deaths in Europe 15. Without further measures, some believe that the number of annual deaths worldwide may rise as far as 10 million, possibly causing the next pandemics 13.

Misuse of antibiotics plays a key role in the development of bacterial strains resistant against this medication. Yet to understand that antibiotics do not work against SARS-CoV-2 and viruses in general, one needs to know that viruses have no metabolism these substances could interfere with. Thus, fundamental knowledge is needed for informed decision-making in health issues. Where else than at school could this foundation be laid? Consequently, we believe that it is not only the health-care side which has to be made aware of the problem as suggested by Little et al. 16 and Tonkin-Crine et al. 17. As interviews with general practitioners have shown, very often patients insist on antibiotics prescription:

“Antimicrobial resistance is beyond the surgery's control a lot of the time because it is patient expectations (…) the patient insists and insists and that's not the clinician's fault that antibiotics are prescribed in the end.” (18 p. 4)

Therefore, this issue demands full attention in the education of young people, which means educating potential future patients. This is even more important in countries, where antibiotics are available without prescriptions 9.

In the work presented here we aimed to analyse virus-related knowledge among samples from i) the general Austrian population and ii) Austrian students from secondary school. Our main research questions were:

- Is the virus-related knowledge of the general population significantly better than that of students from secondary school (both generally and compared with students in upper secondary, when virology has a more prominent place in the curriculum)?
- Within school, can a significant gain in virus-related knowledge be observed along grades?

Two hypotheses were hold equally likely for question 1: a) Adults, who are confronted with the pandemic in a much more comprehensive way than children (family, job, responsibility for taking decisions such as whether getting vaccinated or having their children vaccinated etc.), may try to keep informed in more detail than children and teenagers, who may suffer from distance learning and contact restrictions, but otherwise do not have to take responsibility for further decisions. Second, adults will generally have more experience in classical media consumption (e.g., reading newspapers, watching news), while students may often rely on very short information chunks conferred in social media. Thus, the knowledge levels of adults may be higher. b) Students, especially upper secondary ones, may have been confronted with well-prepared up-to-date knowledge at school, if their teachers used the pandemic to place more emphasis on virology in biology lessons. In this case, at least students in their final years at school may equal or even surpass those, who had left school several years ago and had to try to grasp the complex nature of virology-related topics on their own. Thus, the knowledge levels of (older) students may be higher.

For question 2, we expected an increase of knowledge with grade, but differences between school types, since upper secondary schools in Austria (especially the AHS (= gymnasium), which is the main school type leading to general A-levels) often teach natural sciences in much more detail also in their lower grades compared to middle schools, which only offer lower secondary teaching.

A third research question was related to the kind of knowledge asked for:

- Is there any difference between knowledge directly related to Covid-19, which could easily have been acquired from the media, and more general knowledge on viruses and knowledge related to other viral diseases?

Here, our hypothesis was, that the level of knowledge concerning information about Covid-19 and SARS-CoV-2 that was presented in the media almost routinely would be much higher than knowledge concerning general virus-related topics, which would have required more active information research and processing.

Since vaccination has been a highly controversial topic, we were also interested in knowledge about and attitude towards vaccination against Covid-19 and other viral diseases. Here, our fourth research question was:

- What kind of knowledge and attitude do study participants exhibit with relation to vaccination?

Here, our hypotheses were a) that attitudes would differ highly among participants, but may possibly be influenced by virus-related knowledge, and b) that general knowledge about vaccines, vaccination and vaccination damage may be rather low due to the complex nature of the issue.

Consequently, knowledge in this work was divided into three domains: knowledge about Covid-19, knowledge about viruses in general, and knowledge about vaccination, combined with attitudes and interest items. These domains were studied with a variety of items testing knowledge related to very different facets of each domain.

The underlying rationale was to find out which knowledge gaps and potentially problematic attitudes may need to be addressed at school to give advice to teachers where to place more emphasis on. This paper may thus be understood as diagnostic concerning which virology-related topics have to be dealt with at school in more detail.

### Knowledge about Covid-19 and attitude towards protective measures

Much has been done to find out what people know about Covid-19 since spring 2020. The proportion of people in the respective study groups being well-informed about Covid-19, its causal agent and transfection routes as well as measures to prevent infection has been relatively high, even in studies conducted during the first phase of the pandemic. This has been shown for, among others, Saudi-Arabian nursing students 19, adults from Nigeria and Egypt 20, Chinese adults 21, U.S. adults 22, 23, 24, UK adults 24, and Portuguese university students 25. On the other hand, a strong minority in many studies believes in myths such as that the virus is a hoax or man-made (e.g., nationwide surveys from UK 1 and U.S. 26). Additionally, an important minority and even several physicians or other health care staff seems to believe that this viral disease can be treated with or even prevented by antibiotics 24, 27, 28, 29, 30.

Some studies indicate that certain demographic parameters seem to be strong predictors for high Covid-19 related knowledge and awareness of responsible behaviour, e.g., education and income, age, certain political views as well as type of information source (with the latter, in turn, influenced by knowledge and beliefs) 1, 20, 22, 23, 26, 29, 31.

Yet although there is a wealth of information available about people's knowledge on Covid-19, vaccination, and measures to prevent spread of viral diseases from various nations and population sub-groups, astonishingly, there are almost no data as to the knowledge, beliefs and attitudes with respect to these topics among school children. Yet, as has been argued elsewhere 32, 33, it is at school where most members of a population may learn about these topics. If we provide children and teenagers with sufficient knowledge and the ability to critically analyse information sources, they may be well-suited for informed decision-making in such health-related issues. As results indicate, such foundation may be even more important when the number of information sources reaches figures beyond individual manageability, making assessment of individual sources very difficult 34. Okan and colleagues call this “infodemic” (34 p.1ff.).

### Knowledge about other viral diseases and viruses in general

Covid-19 is by far not the only viral disease causing severe and possibly long-lasting damage to health. Yet studies concerning cervical cancer caused by human papilloma viruses (HPV), ebola, zika, or influenza all show that knowledge about viral diseases is often highly fragmentary - with the exception of AIDS caused by human immunodeficiency virus (HIV), which is an obligatory topic in many countries' school curricula 35, 36, 37, 38, 39, 40, 41. In a study conducted with Austrian high school and university students, several participants wrongly named bacterial diseases or even malaria, when asked to list as many viral diseases as they could recall 8. Apparently, the understanding of the causal agent for a specific disease is not well-grounded. But, as has been explained above, a thorough foundation of virus-related knowledge including structure, means of replication, hosts, diseases and, generally, differences to bacteria and other organisms is pivotal to allow people to come to well-grounded health-related decisions such as when and why to take antibiotics or vaccines.

### Knowledge about and attitude towards vaccination

Viruses cannot be destroyed by antibiotics and presently there is no real cure for viral diseases (with the exception of hepatitis C) apart from the highly expensive treatment with antibodies against, for example, SARS-CoV-2. Mostly, available medication can only halt disease outbreak in the body by hindering virus multiplication or host cell infection (e.g., in the case of HIV). Consequently, vaccination is the method of choice to prevent the spread of viral diseases; all the more, since several vaccines offer lifelong protection (e.g., against the measles). Yet acceptance of vaccination seems to depend highly on whether the vaccine is part of a general (and usually obligatory) state vaccination program, whether it is recommended by health care personnel, in particular general practitioners, and whether the vaccine is on the market for a period deemed long enough, so that potentially negative side-effects would have become public. Mostly, barriers to acceptance of vaccination were due to concern with respect to safety and efficiency 35, 42, 43, 44 or because vaccination in general or at least the booster dose was regarded as unnecessary, even among health care personnel and school teachers 42, 45. Nevertheless, even government recommendations may remain unheeded 46.

Furthermore, there are many myths concerning vaccination, e.g., that they may cause autism - a belief even endorsed by a strong minority of medical students and health professionals 47, 48. This, in turn, may contribute to vaccination refusal 49. Then, the way vaccination works is unclear to many 50. Thus, in a recent study from Britain, about 15% of interviewees showed strong Covid-19 vaccination hesitancy, while this was positively correlated with coronavirus conspiracy and vaccine conspiracy beliefs 2. Concerning young people, some studies indicate a relatively high vaccination knowledge among teenagers 44, 51, others report the contrary both for adolescents and university students 8, 37.

Clearly, education plays an important part in knowledge about available (and/or mandatory) vaccines, and it may also influence attitude toward vaccination 2, 46.

## Methods

We performed two cross-sectional studies with online questionnaires: Study A was conducted Austrian-wide and open for everyone but rather addressing adults. Study B specifically targeted pupils from the three Austrian states Styria, Tyrol and Burgenland. These states had been chosen, because the respective regional school authorities had granted their permission timely. Items for the questionnaire were based on several studies 8, 38, 40, 52, 53, 54, 55. For example, the drawings among which participants had to identify viruses were stylized representations of drawings students had provided in Simon et al. 8. The questionnaire was piloted in several steps: First, all items were validated in that sample questionnaires were filled out by five persons (one male and four females; four between 20 and 30 years, one between 40 and 50 years) with pedagogical, scientific or sociological background, respectively to test whether answering patterns were according to expectation. Second, items were discussed with a further six persons (two males and four females; three between 20 and 30, three between 40 and 50; three with and three without academic background). These validation steps prior to the main study were performed to minimize the possibility that participants misunderstood phrasing and to check whether answers were within the range of expectation.

As explained above, items were grouped into three knowledge domains: i) knowledge about Covid-19/SARS-CoV-2, which was easily accessible due to intense media coverage of the respective items; ii) knowledge about vaccination; iii) knowledge about viruses in general. Both of the latter focused on knowledge which was not generally covered by the media, but most of which should have been taught in school, at least in upper secondary.

Since items even within these three domains tested for different facets of knowledge, we decided against Cronbach's alpha as a measure for reliability. Instead, we performed a re-test reliability calculation: For determination of retest-reliability, the online version of the final questionnaire was distributed to people personally known, but not related to the first author to ensure a wide range of educational background and age and to increase the likelihood that the questionnaire was taken seriously. At the first date (R1, Oct. 15^th^, 2020) 53 persons participated, of which 49 filled out the questionnaire again two weeks later (R2). Within this second survey, items were presented in different order to minimize routine answering due to the short time-period in between (the same individuals answered the questionnaire two times). Five questionnaires of R2 could not be assigned due to unclear codes participants had provided. Thus, data from 44 participants were used to calculate retest-reliability. 52.3% were female and 47.7% male. 52.3% were between 21 and 30 years old, 22.7% between 31 and 40 years, and 13.6% between 41 and 50. Only one was younger than 21 and four people were older than 50. Exactly half of the participants had A-levels as their highest degree, 31.8% GCSE and 13.6% a university degree. 93.2% said that they had no job-related virus knowledge. 31.8% were interested in viruses. Retest-reliability was deemed acceptable (Table S1/A). However, items about knowledge regarding the SARS-CoV-2 virus only had reliability values of 0.62, possibly, because at the time the R-test was performed new information concerning this virus was presented in the media almost daily. This might have influenced participants in their answering.

The final questionnaire was distributed as a limesurvey through a variety of channels, including the Austrian Press Agency, print media, the webpages of several Austrian newspapers and the Austrian State Radio and Television (ORF) website, and various social media. The survey was accessible for 18 days and ended Nov. 27^th^, 2020, because results had to be presented at the European Researchers' Night this day. It comprised single- or multiple-choice questions, but also contained scaled answers. In some cases, participants were asked to voluntarily provide an explanation for their choice. The final version of the questionnaire contained 29 closed items (some nominal, some ordinal, some single-, some multiple choice). For legal reasons, the youngest age class included in demographic parameters for study A was 14-20, because data protection regulations would have required permission from parents for those below 14 years of age. However, since the survey for study A was distributed in media usually accessed by adults, we assumed that only few students from school would participate in study A.

The questionnaire for study B was almost identical. However, the target group now specifically comprised students from middle or high school. Furthermore, we were interested in comparing results between the three Austrian states participating. Consequently, the item asking for job-related virology knowledge was omitted, and students were asked to tick off which kind of school and which grade they attended, and in which district they lived. Additionally, the items *“Do you feel that you have sufficiently been informed about viruses at school?”*, *“Would you want to be better informed about viruses by media and/or politics?”* and *“Which kind of information would you want from media and/or politics?”* were replaced by *“How well do you feel informed about viruses through your lessons at school?”* and *“About which topics would you like to get further information?”* Furthermore, we were interested in learning whether the questionnaire might stimulate or decrease interest in virology, since this could be important for teachers planning lessons on viruses. Therefore, one scaled item at the beginning asked for interest, a second item at the end with the same scale asked how exciting students found viruses. (We decided for slightly different phrasing to avoid routine answering.) A sample questionnaire can be found at supplements (Survey S1). The link to the limesurvey for study B was sent out to all middle and high schools in Styria, Tyrol and Burgenland specifically addressing biology teachers. The survey ran from midst December 2020 to February 5^th^, 2021 (end of term).

We also conducted a second retest-reliability check since the target group was now much younger. This test was performed after the main study, because we wanted to additionally use these data for analysing, whether student knowledge in this sub-sample would outperform earlier results from their colleagues, because answering would take place later in the school year. Thus, several biology teachers were personally asked via email to have their classes fill out the survey in class two times with at least two weeks in between. Students were asked to not use any help from others or the internet. Teachers were asked to not deal with the topics of the survey during the test phase. Instead, participating classes were told that they would get the results once the survey was finished. Due to Covid-19 restrictions at school (e.g., classes were first taught online only; later, personal teaching of small groups was allowed), we had to let this R-test run from early spring to early summer 2021 to gain sufficient data. However, frequent change in group size due to varying Covid-19 restrictions issued by the government made it very difficult to have the same students fill out the survey twice. Thus, from the original 162 students who had filled out the survey during the first round of the reliability test, data from only 64 students could be used from the second round. From these, 56.3% were female, 39.1% were male and 4.7 “other”. 76.6% had German as first language. Grade ranged from grade 7 to grade 10 with grade 8 most prominently present with 39.1%. 92.2% of students were from high school.

The lower values (Table S1/B) compared to the reliability test for the Austrian-wide study could be due to the fact that a) the period between the two tests was much longer, thus more knowledge could have been acquired in between, and b) that student motivation in an already challenging situation (e.g., frequent change between distance learning and learning at school, difficulties of many teachers to get through the topics laid down in the curriculum) may not have been very high. Support for the first point comes from the fact that knowledge concerning the domain “coronavirus”, but not for the other domains significantly increased by participants from R1 to R2 (p = 0.040) (Welch-F(2, 121.609) = 3.045, p < 0.051, ⴄ^2^ = 0.010). Significant differences between student scores from the reliability test and those from study B (only high school grades 7-10) were noted for the domain “viruses”. Here, participants from study B scored about 0.5 points better than those from the reliability test. However, since the sample sizes of the two study populations were so different, comparability between their results is highly limited.

For data analysis, a self-created scoring system was used to generate an overall score for each participant. In short, points were given according to correctness of answers in a way that both the items themselves and the three knowledge domains the items were grouped in (“Coronavirus”, “Vaccination”, “General virus-related knowledge”) were +/- equally weighed (Table S2). Data were then analysed with SPSS. Influence of the various demographic characteristics was tested using independent t-tests as well as analyses of variance (ANOVA). Before testing, Kolmogorov-Smirnov and Shapiro-Wilk tests were used to determine normal distribution of data. Levene´s test was used to analyse heterogeneity of variance. Specific post hoc tests were performed with Hochberg´s GT2 test in case of very different sample sizes and Games-Howell in case of unequal variances. Estimates of effect sizes are given as Cohen´s d (d), eta-square (ⴄ^2^) as well as Spearman´s Rho (p_s_). All statistical tests were performed with α = 0.05 (two-tailed). Multi-level analyses were not performed, since obligations from our university's data protection office precluded us from collecting data on specific groups such as individual schools or even classes. Missing data were excluded from our analysis, even though this might have introduced further bias. Yet items concerning demographic data had been placed at the end of the survey, which meant that all surveys not filled out completely could not be related to demographic parameters.

**Figure 1:**
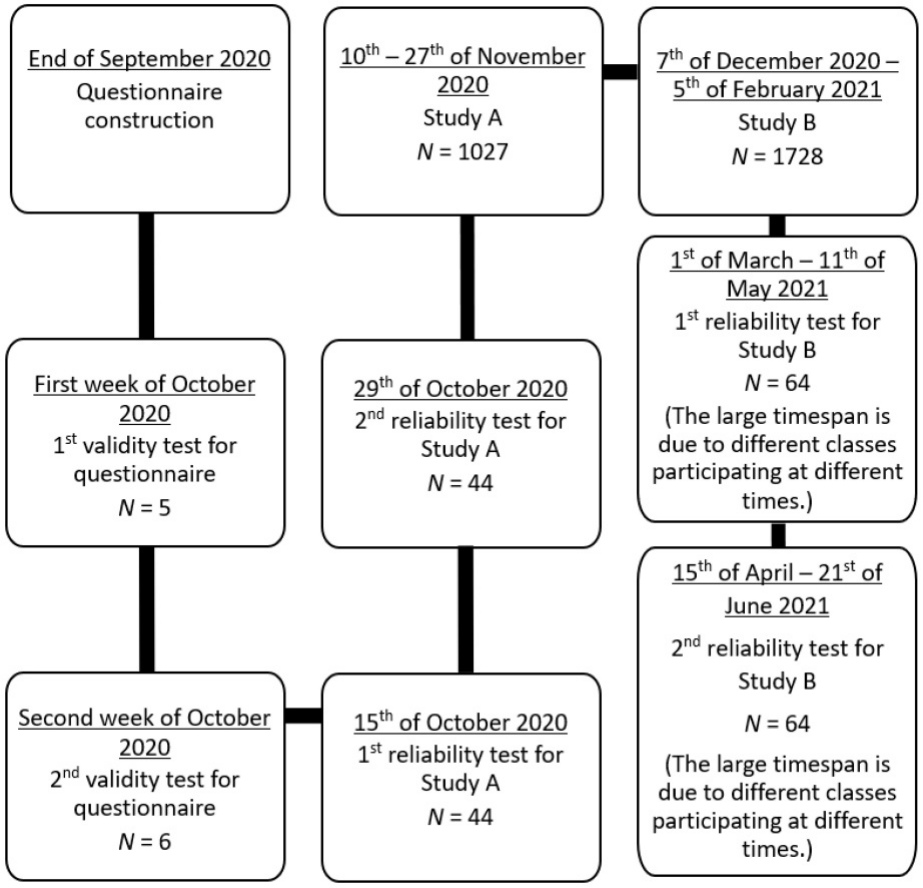
Timeline of studies A and B.

Figure 1 shows the timeline of the work presented here.

## Results

In this section, results are presented in thematic order: First, demographic data from participants are listed. Second, data for the three main knowledge domains (coronavirus, vaccination, general knowledge concerning viruses) are displayed for study A (Austrian population-wide study) and study B (students from grades 5-12/13 = secondary school). Third, attitudes, beliefs and issues participants required further information about are presented. Due to the plethora of data, only selected results are presented.

### Demographic data

In total, 1445 people participated in study A. However, 418 data sets had to be excluded due to incompleteness, resulting in 1027 data sets used for final analyses (58.5% female, 39.5% male, 1.9% “other”). Participants came from all Austrian states with about half of them living in the two biggest cities Vienna (24.5%) and Graz (23.3%). 83.5% reported to have had no prior knowledge about viruses. Of those who had (16.5%), 36.7% worked in health care and social jobs, 35.5% in jobs related to natural sciences and 18.3% in education (Table 1).

In study B, 2305 students participated. Since 577 data sets were incomplete, only data from 1728 participants were used for final analyses (59.7% female, 37.3% male, 3% “other”). Of those, 67.2% were from Styria, 19.2% from Tyrol and 13.7% from Burgenland, which roughly mirrors the relation of population sizes of these states (with a slight bias towards Styria, probably due to Graz being the second largest city in Austria). 83.3% had German as their first language. Other first languages were Turkish (2%), Croatian (2.37%), Bosnian (1.9%), Albanian (1.15%), Rumanian (1.27%), Serbian (0.86%), Hungarian (1.04%) or others (6.11%) (Table 1).

### Knowledge about SARS-CoV-2

**Figure 2:**
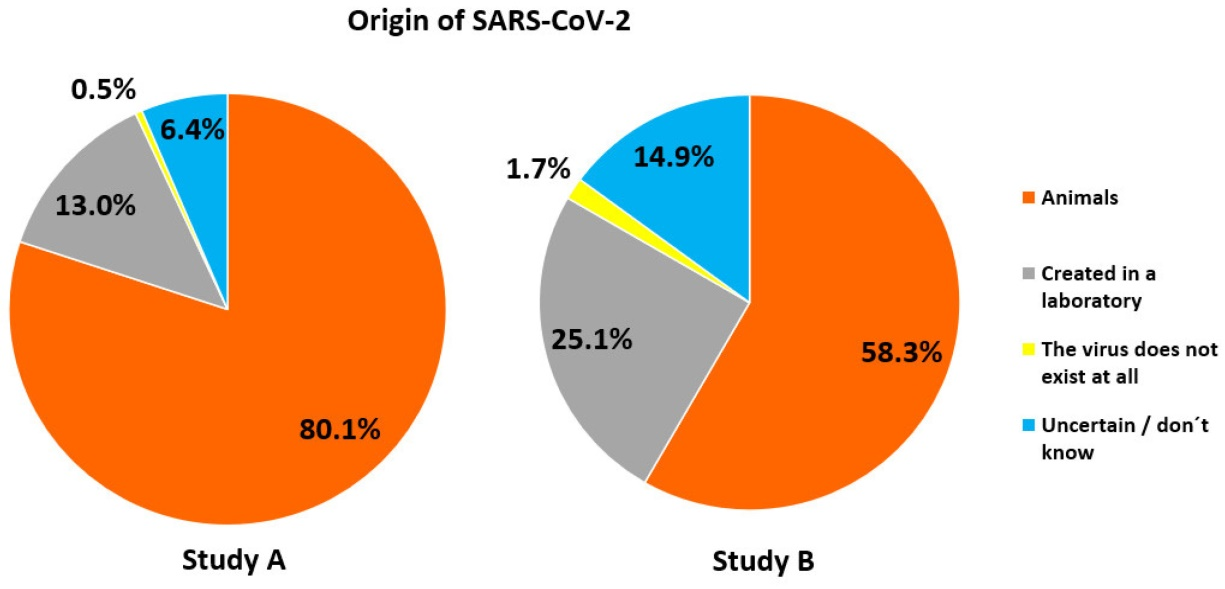
Views about the origin of SARS-CoV-2.

**Figure 3:**
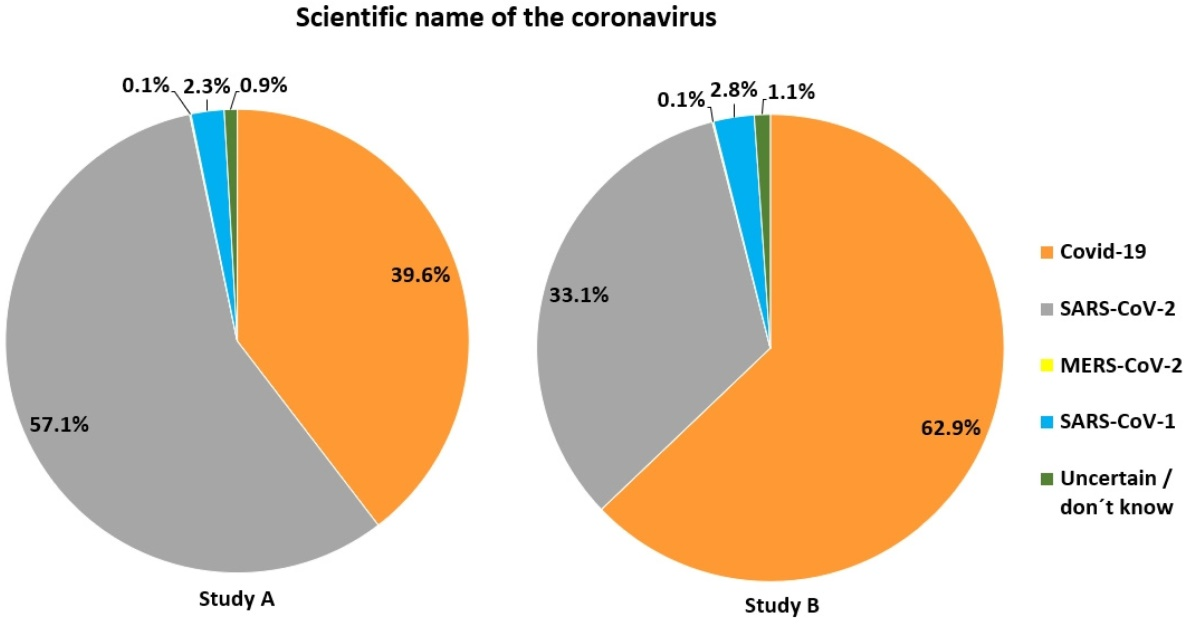
Knowledge of the correct name of the disease caused by SARS-CoV-2.

With respect to origin of the pandemic, 80% of participants in study A correctly stated, that the source of the first human SARS-CoV-2 infections had been animals, while 13% believed that the virus had been created in a laboratory, and 0.5% did not believe in the existence of the virus at all (Figure 2). Furthermore, the majority correctly stated that the scientific name of the virus is SARS-CoV-2 (57.1%), while 39.6% mistook the name of the disease (Covid-19) for that of the virus (Figure 3). For both items, correctness was much lower in study B (Figures 2 & 3).

Concerning transmission of the virus, a great majority in both studies correctly believed that the coronavirus is transmitted through droplets (study A: 89.6%, study B: 78.7%), while all correct options (droplets, aerosols and contaminated surfaces) were only chosen by 42.4% in study A and 15.6% in study B. Similar figures were obtained for children as possible infection source: Here, 62% in study A and 53.2% in study B were certain that children could pass on the virus to same-age peers, while 77.8% in study A and 69.6% in study B thought that adults could be infected through children (both of which is true).

In both studies, the vast majority knew how to test for Covid-19: In study A, 90% and 91.5%, respectively, referred to nasal smear and throat swab, while blood analysis was chosen by 23.7%. In study B, figures were only slightly lower with 89.2%, 84.4% and 19.4%.

**Figure 4:**
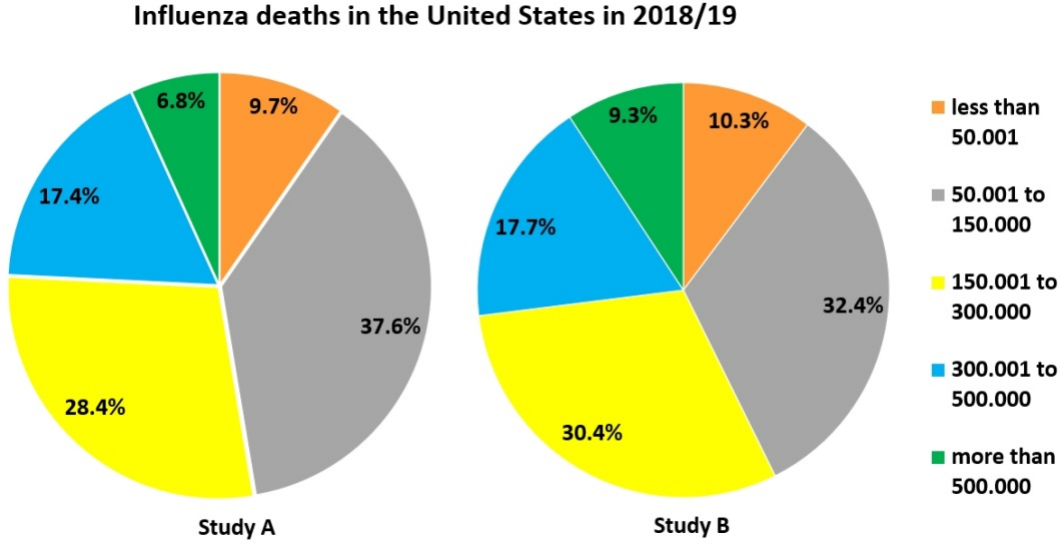
Estimates of how many people died in the season 2018/19 due to or with influenza in the U.S.

**Figure 5:**
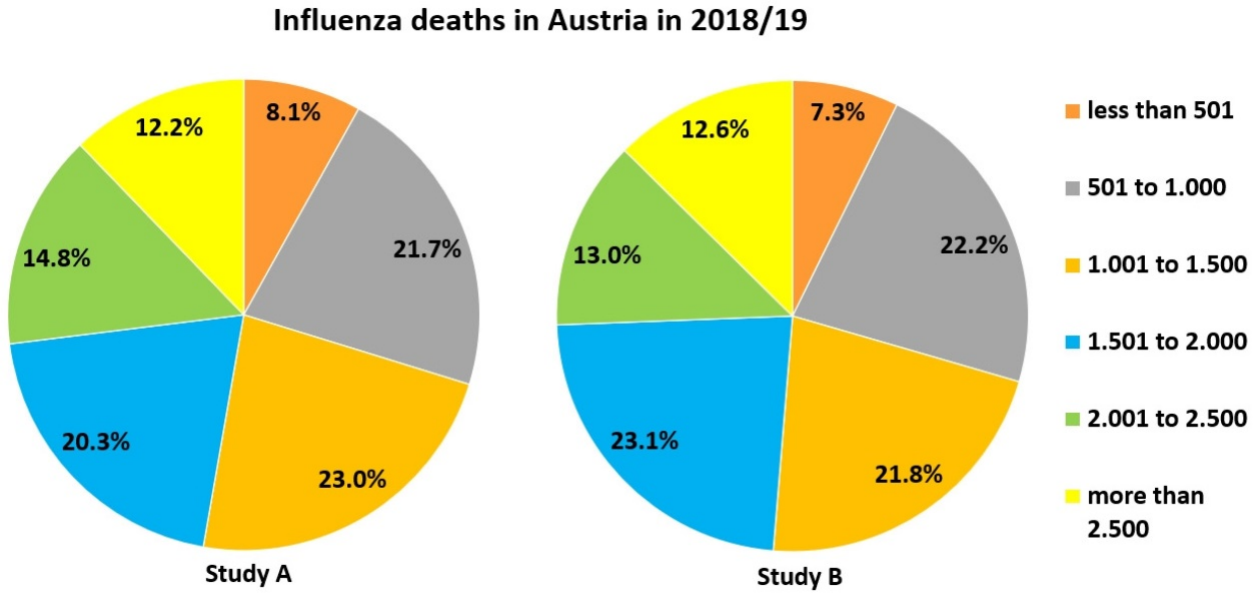
Estimates of how many people died in the season 2018/19 due to or with influenza in Austria.

We were interested in participants' estimates of the danger of influenza, since there is the widespread belief that SARS-CoV-2 is not or only slightly more dangerous than the common flu. Only about 10 % in both studies correctly opted for “less than 50,001“ as the U.S. influenza death toll in 2018/19 (Figure 4), and only about 20 % for “1,001 - 1,500“ concerning the same season in Austria. A large fraction of participants in both studies overestimated the influenza death toll for both countries, but more so for the U.S. (Figures 4 & 5). In fact, approx. 34, 200 people died from/with influenza in the U.S. 56. 1, 373 influenza-associated death cases had been counted in Austria for the season 2018/19, the last before the Covid-19 pandemic 57.

On average, participants in study A gained 3.42 points (SD 0.9) in this domain, thus 62.18% of the 5.5 points maximally possibly (see Table S3 for more detailed analyses). In contrast, students in study B gained, on average, 2.8 out of 5.5 points in this domain (50.9%, SD = 0.97) (see Table S4 for more detailed analyses).

### Knowledge about vaccination

In both studies, most participants were sure that vaccination is used against viral diseases (study A: 90.8%, study B: 80%). However, only 43.6% in study A and 40.7% in study B thought so concerning bacterial diseases even though many recommended vaccines in Austria offer protection against specific bacterial pathogens such as *Clostridium tetani* (tetanus), *Corynebacteria diphtheriae* (diphtheria) or *Bordetella pertussis* (pertussis). Regarding content of vaccines, in study A 70% chose the option that vaccines may contain attenuated forms of a pathogen. 61.1% believed that vaccines contain antibodies, 54.9% that they contain inactivated pathogens. 9.2% believed that antibiotics are present in vaccines. In study B, antibodies were chosen by 63.3%, attenuated pathogens by 50.5% and inactivated pathogens by 26%. 15.3% believed that antibiotics can be part of a vaccine. Thus, a much larger fraction wrongly held antibiotics to be a component of a vaccine in study B.

For the fictitious influenza case study (possible reasons for falling ill despite having been vaccinated in the previous year), 87% correctly voted for mutations of the virus. 65.8% chose the second correct option that vaccinations may not work in every single case, and 12.7% believed that the dose given in the previous year had not been sufficient. 5.5% thought that a vaccination guarantees full protection against a specific disease. For study B, these figures changed to 68.6%, 49%, 16.8%, and 11.2%, respectively.

**Figure 6:**
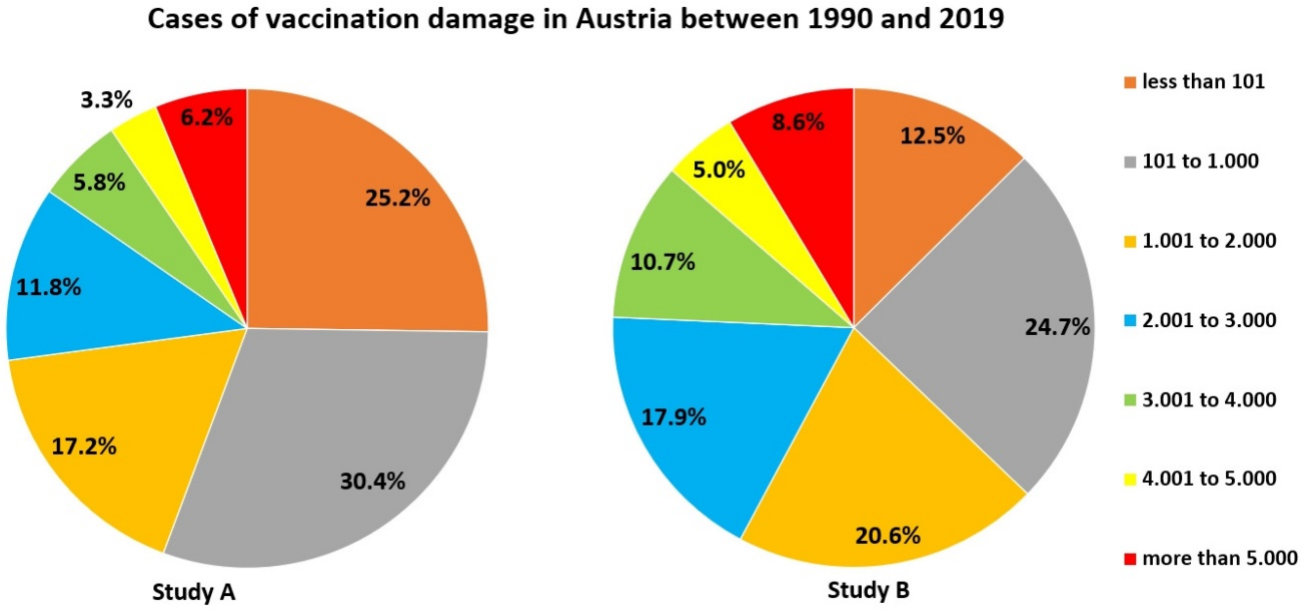
Estimates of how many people experienced vaccination damage in Austria between 1990 and 2019.

44.4 % of participants in study A and two third of participants of study B overestimated vaccination damage in Austria between 1990 and 2019 (Figure 6) (exact number: 409 58).

**Figure 7:**
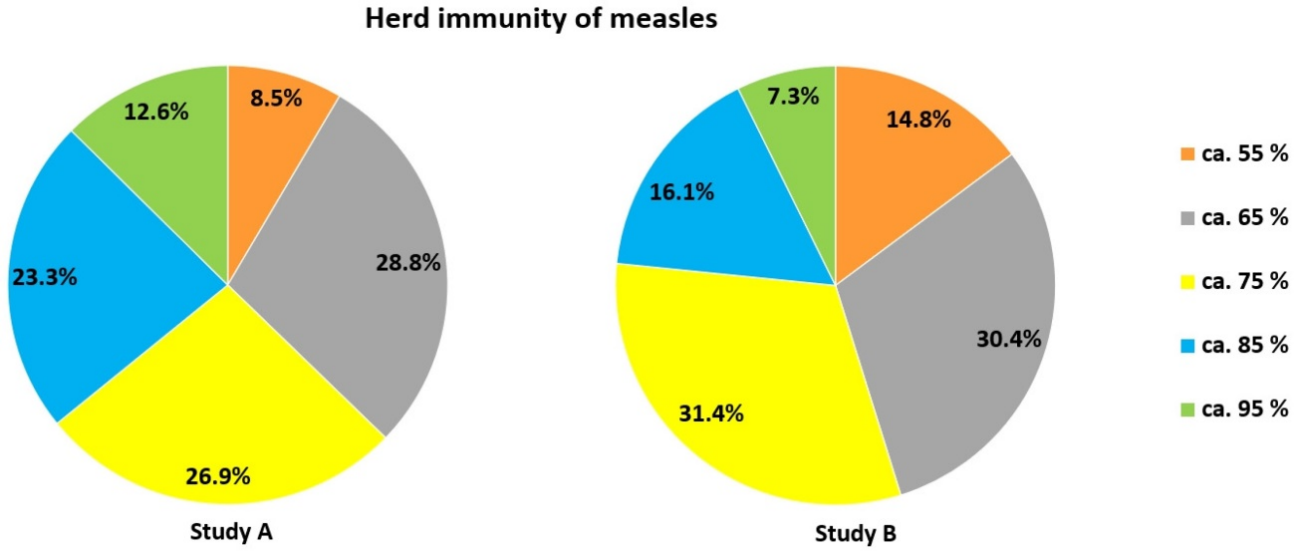
Estimates of when herd immunity would be reached for measles.

With respect to herd immunity concerning measles, only a very minor fraction of participants in both studies gave the correct answer that about 95% of a population need to be immunized: 12.6% in study A and 7.3% in study B (Figure 7). In Austria, only the age group 10-18 yrs. currently meets this goal 59.

In total, the mean score for this domain was 2.45 out of 4.5 points for study A (54.44%, SD = 0.86) (see Table S5 for more detailed analyses) and 1.87 points (41.55%, SD = 0.87) for study B (see Table S6 for more detailed analyses).

### Knowledge about viruses/viral diseases

When it came to more detailed virological knowledge beyond Covid-19, many participants in both studies exhibited severe misconceptions. For example, 27.5% in study A defined viruses as unicellular organisms, about 10% as a kind of bacterium, 36.9% as microorganisms, and 5.6% believed that they are killed by antibiotics. The correct answers (”pathogens” and “non-living particles”) were chosen by 78.6% and 22.9%, respectively.

Since students from middle school (and some from high school) may leave school after grade 8, while others will attend school for 12 or 13 years (A-levels), results are presented in more detail here for these grades, because a fundamental understanding of the virus structure when leaving school is one central issue of this paper as has been explained above. Students from grade 10 are also included, as viruses are often dealt with in grade 9 in upper secondary school. Table 2 displays how students classified viruses. The kind of combinations chosen differed significantly between grades 8, 10 and 12/13 (χ^2^(675) = 907.985, p < 0.001, Cramers V = 0.242), while this had no effect on correctness: There were no significant differences between the scores each grade obtained (ps ≥ .163).

When asked about differences between bacteria and viruses, 54.3% in study A correctly thought that bacteria were more complex (study B: 37.7%). 31.4% (study B: 40.1%) wrongly believed that viruses were more dangerous for humans than bacteria (this is very much dependent on the individual pathogen). 13.8% (study B: 22.7%) viewed bacteria as smaller than viruses. 11% (study B: 18%, but 23% of grade 8 and 19.2% of grade 12/13) thought that antibiotics would help against both bacteria and viruses. 21.3% (study B: 24%) either did not know the answer or were uncertain (see Table S7 for more detailed results from study B). Here, grade had a significant influence on both the chosen combinations (χ^2^(243) = 363.428, p < 0.001, Cramers V = 0.153) and the achieved score: Students from grade 12/13 (M = 0.12, SD = 0.21) and grade 10 (M = 0.12, SD = 0.24) gained significantly more points than those from grade 8 (M = 0.06, SD = 0.16).

With respect to hosts viruses can be found in, 95% and 94.1%, respectively, knew that viruses can occur in humans and animals in study A. 39.9% believed that they could infect plants, 31.1% said the same for fungi and 24.7% for bacteria. 5.6% did not know the answer or were uncertain. In study B, 84.8% and 83.1%, respectively, thought that viruses can be present in humans and animals. 32.1% thought so of plants (39.7% in grade 12/13 vs. 29.1% in grade 8). 28.8% believed that viruses may found in fungi, but only 22.2% thought so of bacteria. 10.7% did not know or were uncertain. Again, grade significantly influenced answer combinations (χ^2^(405) = 537.918, p < 0.001, Cramers V = 0.186) and score. However, here, only grades 12/13 performed significantly better (M = 1.09, SD = 0.49) than grade 8 (M = 0.92, SD = 0.45, p = 0.001). For example, grade 12/13 students chose the correct combination of all five options almost thrice as often as those from grade 8 (13.8% vs. 4.7%).

Concerning diseases, Table 3 shows the percentage of participants having classified a particular disease as viral. Although results are similar across both studies, numbers were generally much lower for study B. However, almost every fourth student was uncertain or did not know the answer across all eleven items. Students of grade 12/13 gained significantly (p = 0.001) more points here (M = 0.69, SD = 0.66) than those from grade 8 (M = 0.46, SD = 0.52). Generally, even diseases which are covered quite extensively in schoolbooks, some of which like measles even in lower secondary, were often wrongly classified.

How do viruses multiply? In study A, 64.9% correctly opted for transfer of genetic material into the host cell, and 44.4% chose the second correct option that viruses need assistance, since they do not possess an own metabolism. On the other hand, 10.7% believed that genetic material would be transferred to other viruses, and 22.8% thought that viruses replicate through simple splitting, making two viruses out of one, as is true for bacteria. For study B, Table 4 displays how students thought that viruses are multiplied. Again, grade significantly influenced answer combination (χ2(234) = 423.458, p < 0.001, Cramer's V = 0.165) and score. For example, students from grades 12/13 (M = 0.36, SD = 0.41) and grade 10 (M = 0.29, SD = 0.4) reached significantly more points than those from grade 8 (M = 0.18, SD = 0.32, ps ≤ 0.026).

Concerning the question, how the immune system recognizes a virus, 35.1% in study A rightly opted for antigens of the virus, 21.8% for antibodies of the virus, 24.5% did not know the answer or were uncertain. A similar confusion between “antibodies” and “antigens” was noted in study B (Table 5). For this item, too, grade significantly influenced the chosen combination of answers (χ^2^(36) = 91.028, p < .001, Cramers V = .115) and students of grade 12/13 (M = 0.17, SD = 0.24) and 10 (M = 0.14, SD = 0.22) performed significantly better than those from grade 8 (M = 0.06, SD = 0.17, ps < 0.001).

**Figure 8:**
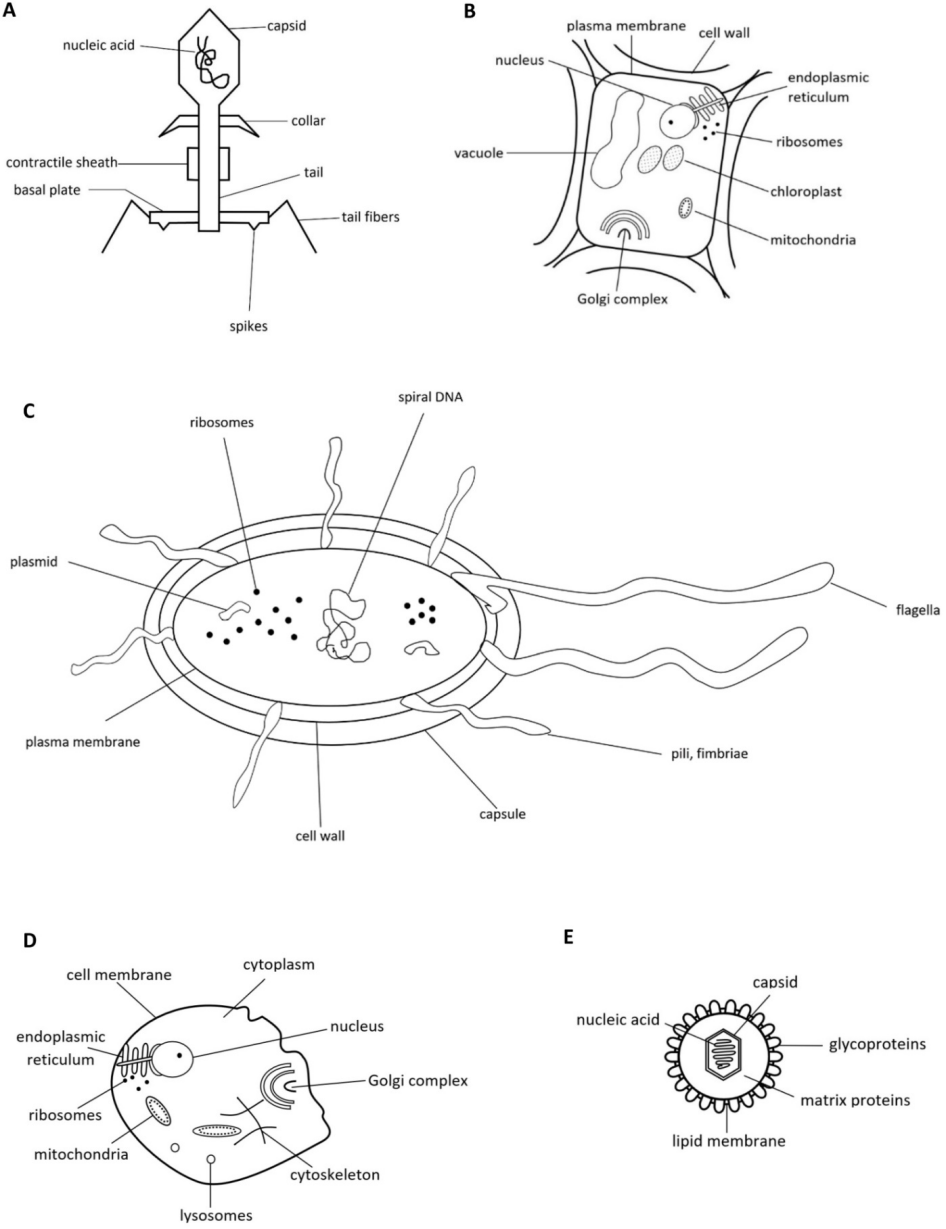
** A.** Stylized drawing of a phage. **B.** Stylized drawing of a plant cell. **C.** Stylized drawing of a bacterium. **D.** Stylized drawing of an animal cell. **E.** Stylized drawing of an enveloped virus. Participants had to tick off which drawings they thought resemble a virus.

When having to identify viruses among the drawings (Figure 8a-e), 48.8% in study A correctly chose an enveloped virus (21.8% chose this as the only answer) and 31.2% a phage (9.2% chose this as the only answer). On the other hand, 34.8% decided for a bacterium (20.6% ticked off this as the only answer), 9.3% for an animal cell and 4.5% for a plant cell. 16.8% combined the two correct drawings.

Students in study B voted most often for the drawing of a bacterium (52 %) and an enveloped virus (33%) (Table 6). Here, too, grade had a significant influence on the combination of pictures students chose to represent viruses (χ^2^(414) = 608.005, p < .001, Cramers V = .198). Grade 12/13 students (M = 0.28, SD = 0.37) and grade 10 students (M = 0.23, SD = 0.32) earned significantly more points than grade 8 students (M = 0.11, SD = 0.25, ps < .001).

The sketch of an enveloped virus was thus chosen by a minority in both studies only, even though pictures of SARS-CoV-2 have dominated Austrian media since spring 2020.

In total, the mean score for this domain was 3.61 out of 8.75 points for study A (41.25%, SD = 1.92) (see Table S8 for more detailed analyses) and 2.44 points (27.88%, SD = 1.4) for study B (see Table S9 for more detailed analyses).

### Total knowledge score in relation to demographic parameters and attitudes/beliefs/interest

*Study A:* The 1027 participants gained, on average, 9.48 points (*SD* = 2.99) of 18.75 possible points (50.56%). Males displayed a significantly higher knowledge level than females. The age group 21-40 performed best. The knowledge difference was greatest between those below 21 and those between 21 and 40, yet it was also significant between those being between 21 and 40 and those being between 41 and 60. With respect to education, those with a university degree performed significantly better than all other groups. Participants with A-levels also reached significantly more points than those with GCSE and without final secondary degree. No significant difference was found between participants with GCSE and without final secondary school grades. Furthermore, people who regarded themselves as equipped with some virology knowledge performed significantly better than those who did not. Persons clearly willing to become vaccinated against Covid-19 (strong Yes) also obtained significantly more points than those who did not (strong No). Participants with German as mother tongue reached significantly more points than those with other languages. Additionally, those being personally interested in the topic scored significantly better than those who were not (Table 7).

*Study B:* Across all three knowledge domains students obtained, on average, 7.11 of 18.75 maximally possible points (37.92%, SD = 2.55). As for all domains separately, there was no significant difference in scoring between males and females and no significant influence of state/location of school. Yet students with German as first language performed significantly better than students with other first languages. Willingness to become vaccinated was also strongly correlated with knowledge about viruses: Those strongly in favour of getting vaccinated scored much better than those strongly opposing their own vaccination. However, no significance was observed concerning mandatory vaccination. Upper secondary students gained significantly more points than those from lower secondary. Grade had a significant influence. For example, students of grades 10-13 scored significantly better than students from grades 5-8. Both students from grade 12/13 and from grade 10 performed significantly better than students from grade 8. School type significantly influenced results: For example, lower secondary high school students achieved significantly more points than their same-age peers from middle school. Students from general education high schools yielded significantly more points than students from specialized high schools (Table 8).

### Comparison of study A and B

**Figure 9:**
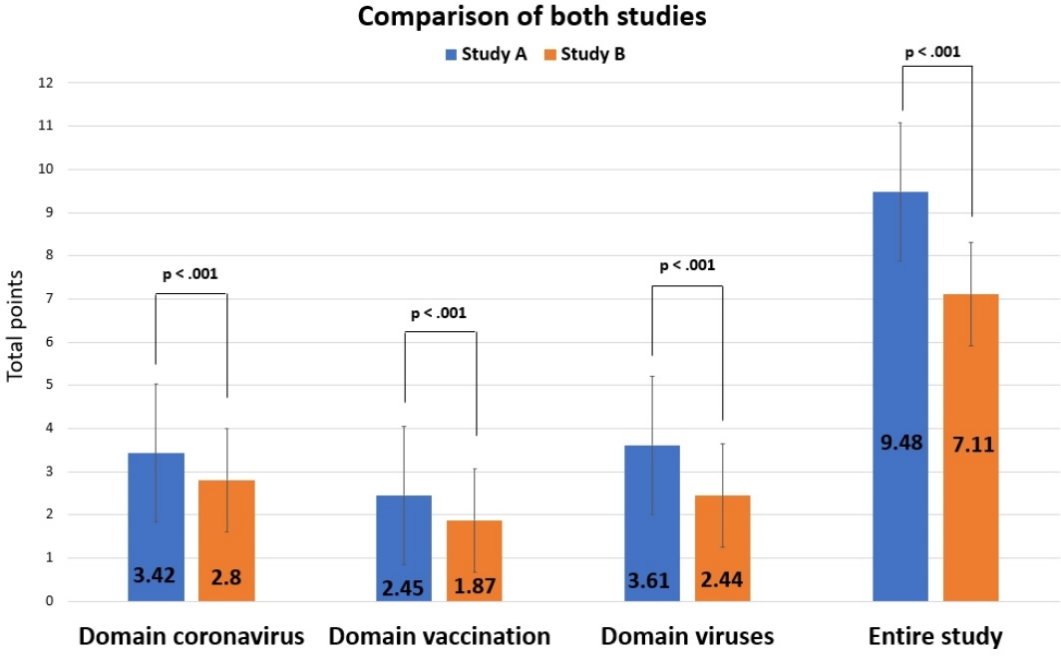
Comparison of results from studies A and B.

The comparison of both studies (Figure 9) shows that knowledge among participants of study A was significantly better both for each domain (coronavirus: *t*(2281.014) = 17.012, *d* = 0.658, 95% - CI for *d* [0.579, 0.737]; vaccination: *t*(2753) = 16.847, *d* = 0.664, 95% - CI for *d* [0.585, 0.743]; viruses: *t*(1677.097) = 16.998, *d* = 0.724, 95% - CI for *d* [0.644, 0.804]; *ps* < .001) and in total: The average score of study A was M = 9.48 (SD = 2.99), that of study B was M = 7.11 (SD = 2.55, *t*(1898.464) = 21.182, *p* < .001, *d* = 0.868, 95% - CI for *d* [0.788, 0.949]).

**Figure 10:**
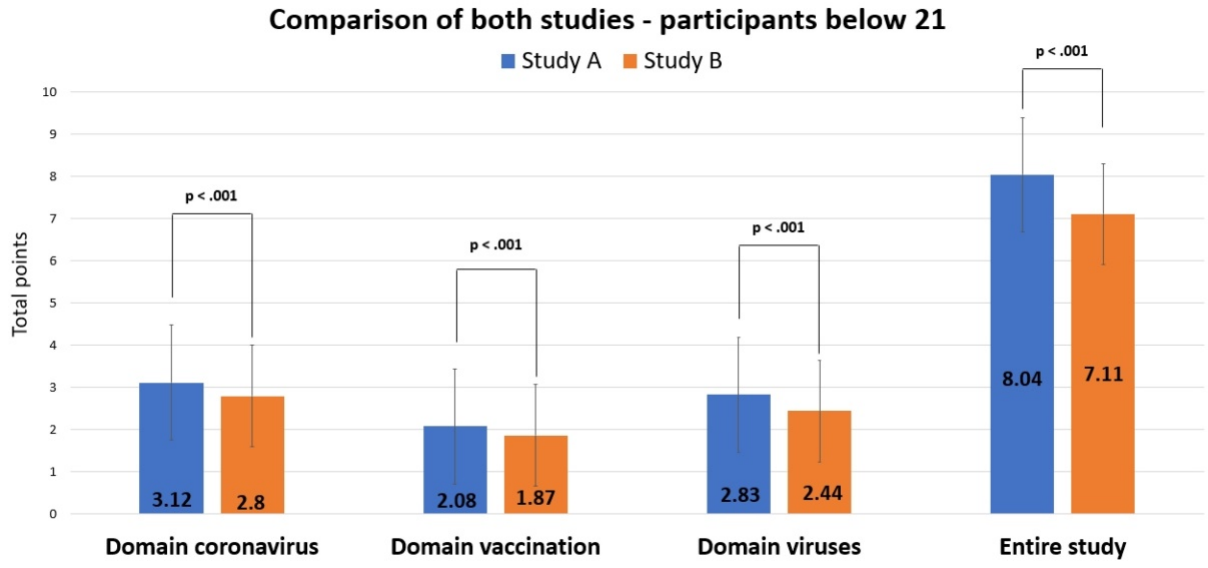
Comparison of results from studies A (< 21 yrs.) and B.

When data from study B (N = 1728) were compared only with the age group < 21 from study A (N = 543), scores were still significantly better for study A for all domains (coronavirus: *t*(978.229) = 7.193, *d* = 0.339, 95% - CI for *d* [0.242, 0.435]; vaccination: *t*(970.404) = 5.205, *d* = 0.246, 95% - CI for *d* [0.149, 0.343]; viruses: *t*(2269) = 5.666, *d* = 0.279, 95% - CI for *d* [0.182, 0.375]; *ps* < .001) and in total (*t*(2269) = 7.497, *p* < .001, *d* = 0.369, 95% - CI for *d* [0.272, 0.466]), though less strong than when including all participants of study A (Figure 10).

### Attitudes/Beliefs/Interest

**Figure 11:**
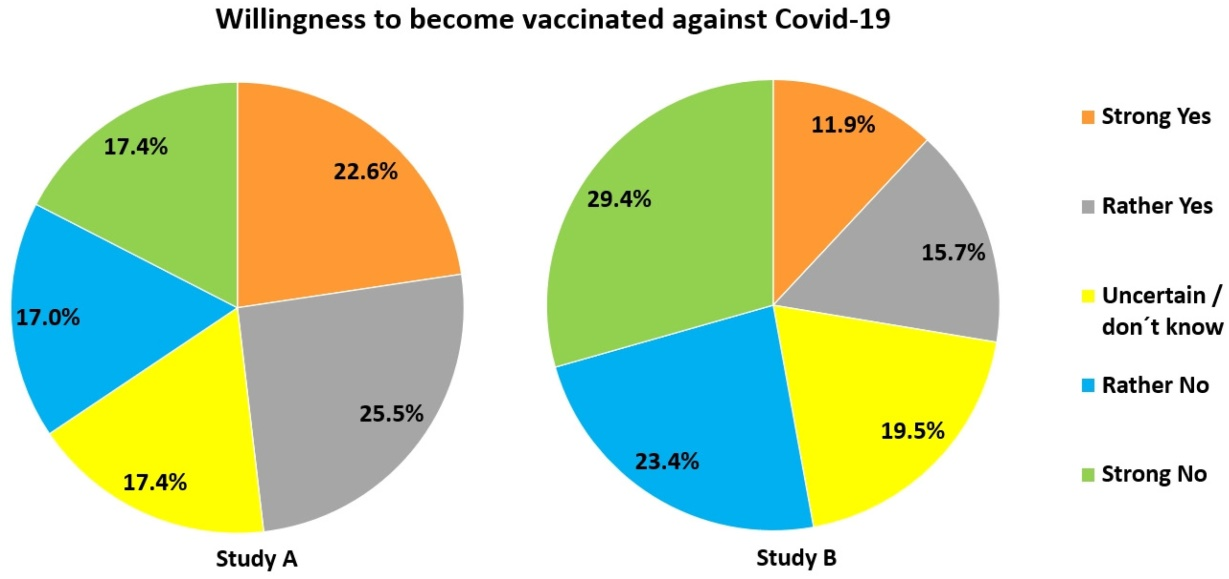
Acceptance of own vaccination against Covid-19.

**Figure 12:**
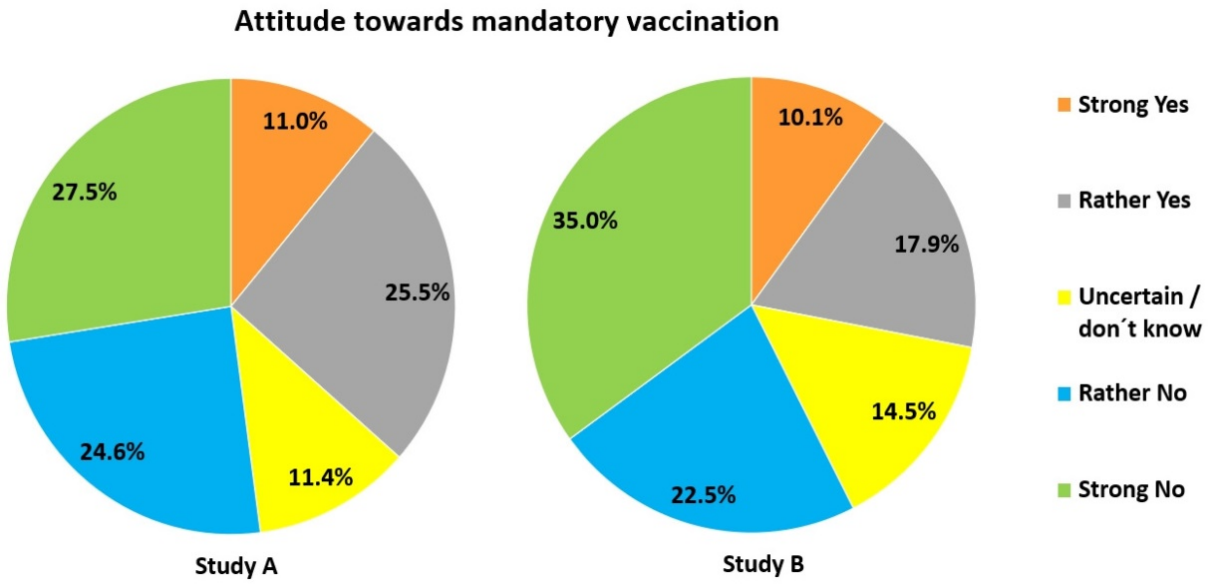
Acceptance of mandatory vaccination against Covid-19.

*Study A:* 55.1% had no interest in virus-related topics. Only 48.1% were willing to become vaccinated against Covid-19. 34.4% refused vaccination (Figure 11). Reasons provided were (in case of Yes): contributing to herd immunity (8%), protecting oneself and others (21.5%), having a high job-related risk of contracting the virus (0.9%), being member of a risk group (1.3%) or being a vaccine supporter (1.6%). Pro-vaccination participants felt that the vaccine was safe (7.6%) (esp., when officially approved) and had confidence in science (1.9%). Reasons provided by those against vaccination were: uncertainty due to the novelty of the mRNA-vaccine (35.2%), or no need for vaccination (5.1%) (because of well-functioning immune system, young age or having already had Covid-19). Some argued that they would presently step back in favour of risk group members (3.3%). 1.6% were vaccine opponents and 0.7% doubted the danger of COVID-19. 4.9% wanted to wait for initial experiences with the vaccine. Mandatory vaccination was far less acceptable (Figure 12).

**Figure 13:**
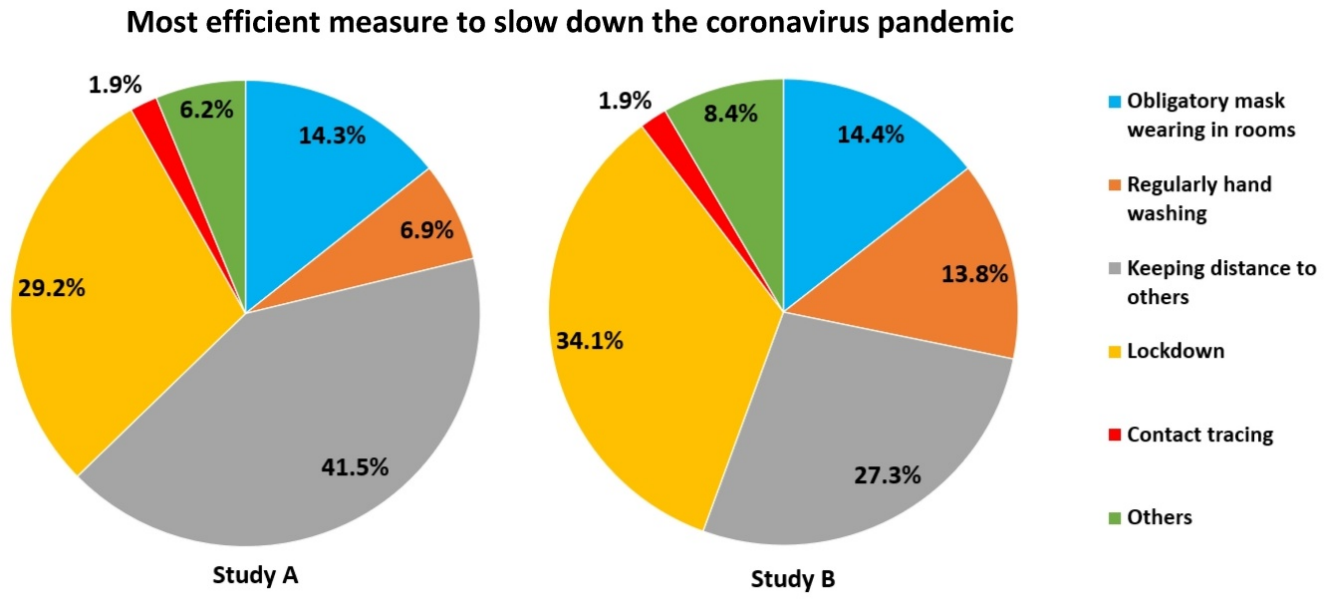
Most efficient measures to slow down the spread of SARS-CoV-2.

When asked which measure would be most efficient for slowing down the pandemic, most voted for keeping distance, lockdowns and mask wearing (Figure 13). Some participants (2.6 %) suggested a mix of various measures.

*Study B:* At the beginning of the questionnaire 73.2% of the students regarded the topic “virus“ as interesting (10.6% very interesting, 62.6% interesting), 26.8% as not (4.6% highly uninteresting, 22.2% uninteresting). At the end, 68.1% found viruses “exciting“ (12.4% very exciting, 55.7% exciting), while 31.9% found it “boring“ (6.6% very boring, 25.3% boring). Thus, several participants changed their view about viruses in the course of filling out the questionnaire: While 72.4% did not change their view and found viruses similarly interesting/exciting at the beginning and at the end, there was a rise in interest for 11.17% and a decrease for 16.44% (z = -4.121, p < .001, n = 1728).

With respect to measures preventing spread of the virus, most voted for lockdowns, keeping distance and mask wearing, but many also for hand washing (Figure 13). Other options provided were “combination of different measures”, “group teaching at school”, “testing”, “vaccination”, “closure of borders” etc. However, 13.5% of those having opted for “other” refused all such measures, but favoured an endemic contamination of the population. 3.5% held such measures unnecessary and regarded SARS-CoV-2 as a hoax.

43.2% felt that they had been “well informed“, 9.4% even “very well informed” about viruses through their teachers. On the other hand, 33.3% considered their learning about viruses at school as “mediocre“, 8.8% as “bad “ and 5.3% even as “very bad“. Students who viewed themselves as well-informed scored significantly better (M = 7.37, SD = 3.16) than those who felt very badly informed (M = 5.88, SD = 2.61) (t(217.187) = 4.045, p < .001, d = 0.501, 95% - KI for d [0.241, 0.761]). The same was true for students who found viruses highly interesting (M = 8.19, SD = 3.07) versus those that had no interest in virology (M = 5.31, SD = 2.28) (t(199.777) = 8.444, p < .001, d = 1.009, 95% - KI for d [0.732, 1.285]).

With respect to vaccination (Figure 11), 27.6% would have let themselves be vaccinated against Covid-19, but 52.8% would not. 19.5% were undecided. The following reasons were given in favour of getting the vaccine: self-protection and protection of others (12%), contribution to herd immunity (7.6%), general trust in the vaccine(s) (2.4%). One participant wrote that he/she had lost a relative through Covid-19. The following reasons were given against vaccination: uncertainty concerning vaccine safety (50.2%), no interest in getting vaccinated (2.8%), no need to do so because of young age, strong immune system or having already had the disease (2.4%). 1.3% said that people from high-risk groups and those relevant for a functioning system should become vaccinated first. 1% regarded themselves as vaccination opponents. 4.2% wanted to wait (further explanation was not provided), 4.3% were undecided. Concerning mandatory vaccination 28% were positive and 57.5% negative (Figure 12).

### Issues participants wanted to know more about or raised separately (open-ended items)

*Study A:* Two thirds of the participants stated that they had not been taught sufficiently about viruses at school and 37.3% wished for more detailed information, e.g., with respect to virus structure, transmission and effect in the body (57.2%), differences between viruses and bacteria (2.7%), and hygiene measures (8.9%). Some demanded a better visibility of experts in the public discourse (4.3%) and a better position of biology in school curricula (0.3%).

*Study B:* 42.6% of the students wanted to know more about viruses and bacteria in general. 12.2% demanded more details about SARS-CoV-2 and Covid-19. 1.2% required more information about the vaccines. 2% thought that there was too little discussion as to how to deal with the crisis. 19% of the answers were not comprehensible. The remaining either expressed no need for further information or did so but did not specify the field they would want to know more about.

## Key findings

- Many participants had difficulties to hold apart viruses and bacteria.
- Many participants had difficulties to visually identify viruses.
- Several participants did not seem to know that antibiotics do not work against viruses.
- Many felt insufficiently informed about viruses at school.
- Participants from study A significantly outperformed those from study B in all three knowledge domains tested.
- The vast majority of students, but less than half of the participants of study A was interested in virology.
- Vaccination against Covid-19 was seen positively by less than half of participants.

## Discussion

Our results show that the virus-related knowledge of the general population is significantly better than that of students from secondary school (research question 1). This is even true when comparing participants from study A (N = 1027) with students in their final year of high school (N= 224) (*t*(360.021) = 5.408, *p* < .001, *d* = 0.368, 95% - CI for *d* [0.222, 0.513]). Apparently, Covid-19, vaccination and general virus-related information taught at school to the younger participants (study B) has not equalled the information gain outside school by the older participants (study A).

Yet some knowledge development was observed, as grade played a significant influence, in particular, when comparing lower and upper secondary school, but even within upper secondary. Furthermore, school type (lower secondary high school vs. lower secondary middle school) played a significant role, as was expected for research question 2.

For research question 3 we found, as expected, that participants in both studies performed much better with items directly related to Covid-19, since such knowledge could easily have been acquired from the media. In contrast, knowledge on vaccination and much more so general virus-related knowledge was much lower in both study populations. Yet not only knowledge but attitude towards vaccination, too, differed widely among participants, and was significantly and positively correlated with virus-related knowledge (research question 4).

Specifically, our study shows that there is urgent need to address the following key issues at school and after:

- differences between viruses and bacteria (and other organisms);
- understanding of the structure of a virus aided by visualization through pictures;
- causal agents of important viral and bacterial diseases;
- awareness about functioning of antibiotics and pathogens/diseases antibiotics help against (and those they do not);
- understanding of the importance of vaccination and a realistic assessment of risks of side-effects and vaccination damage.

### Differences between viruses and bacteria

As in Simon et al. 8, a relatively high proportion of participants from both studies defined viruses as some kind of bacteria (about 10% in study A, about 12% of upper secondary students and about one third of students in the last year in lower secondary in study B). Even more choose a drawing resembling a bacterium when asked how a virus may look like (about 35% in study A, about 43% of upper secondary students and about two thirds of students in the last year in lower secondary in study B). Several choose the animal or plant cell. About one quarter of participants in both studies defined a virus as a unicellular organism. Thus, many participants in both studies were unable to clearly differentiate between a virus and bacterium. Perhaps unsurprisingly, quite a few participants thus believed that viruses can be killed by antibiotics (about 6% in study A, about 10% of grade 8 - 12/13 students in study B), and that viruses grow in number by division of one virus into two (about 23% in study A, about 32% of grade 8 - 12/13 students in study B). Thus, misconceptions found in other studies 8, 38, 52, 60 have been widespread in the population analysed here. Although our figures are far lower than those noted in the last survey of the European Commission for Austria 7, a remarkable number of people seem to think that viruses are at least so similar to bacteria that they can be fought off by similar medication. Furthermore, many participants were unable to correctly classify the causal agents behind the listed diseases. Additionally, several participants did not seem to know what antibiotics are and how they work, as they assumed them to be a constituent of vaccines.

### Differences between viruses and *Plasmodium*/Malaria and understanding of virus structure

As in Simon et al. 8, more than 40% classified Malaria as a viral disease. Further research is needed here to understand the underlying misconception. Possibly, the complex life cycle of *Plasmodium*, which also multiplies in host cells and which is given much space in Austrian biology schoolbooks for its great biological interest, is understood by many as highly similar to the HIV replication cycle, which also has a prominent part in Austrian school biology. It is our clear recommendation to teachers and teacher educators to highlight the differences of viruses to both bacteria and *Plasmodium*. Along this line, students should be confronted much more with pictures showing how all the pathogens are organized and look like. This might help much in distinguishing each pathogen and understanding the differences between them. Concerning visual identification of viruses we had included this item for several reasons: i) as an indicator for the ability to distinguish between viruses on the one and bacteria and other living cells on the other hand (structure); ii) to test whether the widespread occurrence of pictures showing the SARS-CoV-2 virus both in print and online media would be reflected in a high recognition rate of the enveloped virus sketch (which was only partly true, and more so for study A); iii) because we believe that part of the fundamental biological knowledge one should have acquired when leaving school is to know how sample representatives from the five taxonomic kingdoms look like, both in general and in cellular/structural form, for precisely the reason to help understand that this taxonomy means that there are fundamental differences between members of these kingdoms.

### Vaccination

The percentage of immunized people to reach herd immunity for measles was highly underestimated by most participants in both studies. Although these figures may have been distorted due to much lower numbers discussed in the media for Covid-19, it hints at a severe problem possibly true for many countries: Very many people may not see the necessity of becoming vaccinated against measles, knowing that about 90% or even more of their nation has already had this vaccine. However, herd immunity for this highly contagious disease is only reached when at least 95% of a population have been immunized. Thus, high as the numbers of vaccinated people may appear, they provide the impression of false safety for too many. The same may be true for vaccinations requiring a booster dose 42. It remains to be seen whether a similar situation will arise for Covid-19. Furthermore, study A and B show that vaccination damage is highly overrated. This must be addressed at school to overcome possible refusal of vaccination as early as possible. The need for this is emphasized by the fact that a large fraction of our study population either refused Covid-19 vaccination or was undecided, even though for different reasons. In this respect it is important to note that willingness to become vaccinated correlated negatively with estimates of vaccination damage (study A: *r_s_*_ =_ .299, *p* < .001, *n* = 1027, study B: *r_s_* = .273, *p* < .001, *n* = 1728). Thus, the higher a person deemed the risk of vaccination damage, the lower the willingness to get vaccination. Vice versa, it is interesting that particularly older people (> 60 yrs.) performed best in the vaccination domain (Table S5). Very likely, they were most interested in obtaining information concerning vaccination, as they were (and still are) considered the most vulnerable group also for Covid-19. Furthermore, younger students are still under the influence of their parents. Households with adult vaccination sceptics may thus have a direct influence on the view of children living there.

Mandatory vaccination was rejected by a large part of both study populations, similar to earlier results from Reiter-Haas et al. 54 and Moritz-Eberl et al. 61. This may have several reasons: Vaccination is generally voluntary (though recommended) in Austria, the Covid-19 vaccines are very new on the market, and reports about side-effects and possible vaccination damage were often reported about in the media in Austria.

Interestingly, those students participating in the reliability test after study B were much more in favour of Covid-19 vaccination than same-age participants from study B: While only 23.1% and 20.5% of high school students grade 7 - 10 had ticked off “strong yes” and “yes” with respect to willingness to become vaccinated against SARS-CoV-2 in study B, these figures raised to 35.9% and 32.8% for the first and even 46.9% and 28.1% for the second R-test. Similarly, mandatory vaccination was now seen much more positively (54.1% “strong yes/yes” as compared to 28% in study B). Apparently, the ongoing public debate concerning vaccination and the possibility that vaccinated people may expect more freedom in everyday life and in travelling may have influenced students here.

Generally, viruses were far more often referred to in conjunction with vaccination than bacteria, even though the Austrian child immunisation program strongly recommends vaccination against, e.g., diphtheria and tetanus. With respect to passive immunization, it remains unclear, why so many chose “antibodies” - perhaps either because of the frequent naming of this term in the current pandemic or due to real knowledge about this kind of immunization. On the positive side many people knew that the influenza virus has a high likelihood of mutating reducing the protection through vaccination from previous years. At the time of study A, vaccination against Covid-19 was still not possible in Austria. Vaccination started on December 27^th^, 2020, with the mRNA vaccine from BioNtech/Pfizer, but, at first, for adults only. As of December 18^th^, 2021, some Austrian states have begun to offer vaccination for children as young as five years old, 6,546,136 people have had their first, 5,979,894 their second anti-Covid-19 vaccination, and 3,301,473 have received a booster dose 62. This means that in December 2021 about two third of the Austrian population have received at least two vaccination doses.

### Hosts

Although most participants named humans and animals as potential hosts for viruses, less did so for plants, fungi and bacteria. Since fungi are usually not discussed much in Austrian schools, this might not be surprising. This is different with plants and bacteria. While the first virus discovered was a plant virus 63, phages as viruses which attack bacteria are present in many Austrian schoolbooks. Thus, teaching definitely needs improvement here; all the more, since phage therapy might be one important way out of the antibiotics deprivation currently warned against in clinical bacteriology 64, 65.

### Danger of Covid-19 in relation to influenza

Our data show that a significant proportion of participants overestimated the death toll of influenza in the season prior to the current Covid-19 pandemic, but overestimation was much more prominent for cases in the U.S. than in Austria. This might have been due to that knowledge of these figures was probably better for the country participants lived in. Secondly, continuous reporting of the high Covid-19 death toll in the U.S. could have influenced influenza estimates. Understanding that SARS-CoV-2 (particularly, the new mutants) is much more dangerous than the influenza virus and that vaccination against Covid-19 is thus even more important than that against the seasonal flu seems vital to successfully combat Covid-19 in public health strategies.

### Implications for school, influence of demographic parameters and attitude

On average, participants in study A scored significantly better than students in study B. This is most obvious for general knowledge about viruses. Apparently, those interested in virology gained much of their knowledge outside school, either in tertiary education or from the media. This becomes even more clear when comparing results from study B only with those participants from study A who were younger than 21 and did not have any specific job-related knowledge about viruses. This subgroup earned M = 7.99 points (SD = 2.31), while students in study B scored M = 7.11 (SD = 2.55), thus significantly less (t(912.708) = 7.427, p < .001, d = 0.354, 95% - CI (0.255, 0.453)). Yet we believe that every student leaving school needs to have a basic understanding of what viruses are, how to prevent contraction and what to do in case of an infection. Apparently, this is a severe gap in school biology urgently asking for change. Schools need to provide their students with a thorough knowledge about viruses to i) allow for information-based decisions concerning health-related issues in the field of virology and ii) to be able to analyse virology-related information and to distinguish between solid science-based facts and fake news.

Then, one has to understand that science is a process in which new data can lead to a new assessment of a given situation - as we all experience right now in the current pandemic. Thus, a basic understanding of the nature of science including its process of knowledge finding is also essential 66. We therefore totally agree with Miller, who, facing Covid-19 conspiracy theories, wrote: “The medical community should mount systematic efforts around science education beginning in childhood and across the lifetime” (66 p. 2256).

The need for improvement in education is reflected in i) the self-assessment of many participants, who felt that school had not equipped them well enough with the virology knowledge they would presently need, and ii) the finding that there were no significant differences between the three Austrian states participating in the survey when comparing lower and upper secondary grades between same school types. Drastically spoken: No matter, where students had been attending school, their level of knowledge did not differ and was, on average, far from acceptable. Thus, improvement of knowledge acquisition in this highly important field of health education seems to be a nationwide task. Additionally, key aspects of virology (e.g., the differences between viruses and bacteria) need to be taught in lower grades, so that all students are equipped with the knowledge they need for future health-related decisions and information evaluation. In the Austrian middle school, those students having had to repeat a school year may leave school after grade 8, which is the final year of middle school. Others may proceed to higher secondary education or, at least, attend a vocational school for one additional year (usually without much biology). But about 56 % of middle school students leave school altogether after grade 8 or 9 67. Our study has shown that these students know significantly less in this field than students from grade 10. Improving this situation is not only a question of social justice, but also of public health, since a fragmentary or even no understanding of when to use antibiotics, how vaccines work, why they are essential in the fight against certain diseases, and which hygiene measures are to be observed to reduce infection with specific pathogens may be vital to reduce the likelihood of future pandemics.

The finding that grade 10 students performed almost as good as students from grade 12/13 for many items might be explained by the fact that, in Austria, viruses are often dealt with in grade 9 in upper secondary school. Generally, our results show that students of final years from lower and upper secondary school performed better than the respective lower grades. Thus, the fact that virus- and vaccination-related knowledge increased with grade indicates some successful teaching in this respect. On the other hand, most students were far below the maximally achievable score, which, again, points to a high potential for improvement.

Concerning interest there is something to note on the positive side: Very many participants found the topic virus interesting. In study B we had installed an internal control item to test whether interest might be influenced by filling out the questionnaire. Interestingly, most students rated viruses as “exciting” in the end as they had rated them “interesting” in the beginning. However, some students changed their mind during the course of the survey either to the better or to the worse. This might have had two reasons: For some, the survey might have become boring or overtaxing (which might also explain the relatively high dropout rate), or the questions they had to answer met their interest only partly. Conversely, some items may have raised issues they were previously not aware of for others. In total, we find it encouraging, that almost half of the participants in study A and about 70% of those in study B found viruses as such interesting - which will facilitate teaching and learning about this topic.

Several demographic parameters had a significant influence on performance: higher educational level, interest in viruses, German as first language, willingness to become vaccinated and (though only for study A) being male all had a positive impact on achievement as had job-related experience with viruses (for study A). The significant influence of first language may be due to different reasons. Since we did not test reading competence, the reduced score of many with a first language other than German might be due to greater problems in understanding item phrasing. Second, since immigrants attend high school less often, this might also be an educational problem. In any case, future educational programs within and outside school need to target this specific sub-population, so that risks for both individual and public health are reduced by reduced language and educational barriers.

A minor, but strong fraction in both studies believed that SARS-CoV-2 was man-made or a hoax, with twice as many pupils believing this myth. Such conspiracy theories have been noted for other populations 1, 26, 54. Yet in general, Covid-19-related knowledge was relatively high in both study groups. Only the role of children in the pandemic seems to have been underestimated by many. This, however, might have been because during the time of the survey and before, prominent virologists in Germany and Austria differed in their opinion, whether infected (but mostly symptomless) children might spread the virus.

Finally, the way we speak about viruses may have a significant influence on how we view them 32, 33. Even in medical university textbooks viruses are often named micro-organisms, which furthermore reproduce. Yet most scientists presently agree that viruses are non-living particles, which cannot reproduce on their own, but which need a host metabolism for replication. Consequently, they are replicated (although in some cases with the help of viral enzymes). The passive voice and the correct labelling might help students both in secondary and tertiary education to understand the differences between viruses on the one and living cellular organisms on the other hand.

Generally, we believe that teachers at school should at least include the following aspects concerning virology:

- Differences between viruses and bacteria both in structure and metabolism, for this being i) fundamental biological knowledge and ii) the ground to understand why viral diseases need different treatment than bacterial ones;
- Some basic understanding of how viruses enter and leave hosts cells and how they are replicated within for the same reasons;
- Knowledge of the most important viral diseases in the respective country and, if available, their treatment and vaccination against;
- A factual discussion of pros and cons of vaccination to i) show that concerns present among so many are taken seriously, and ii) to nevertheless explain that much of this anxiety has no scientific grounds.

## Limitations

Study A: Our sample covered only a small fraction of the Austrian population. Furthermore, both higher educated and younger people were over-represented in our study, possibly, because this study was advertised mostly in online media. Thus, our study may not be representative in this respect, even though our study encompassed more participants than were included in the European Commission survey 7. Consequently, results from study A may actually be too positive, as one may expect a lower virus-related knowledge among less well-educated people. It could well be that many of the latter were lost during filling out the survey, which might partly explain the high drop-out rate in study A.

Study B: The same may be true for study B, where the drop-out rate was also very high. One possible explanation is that the questionnaire might have been too long and/or difficult for several younger students, who might then have stopped filling out the survey. This could also explain the very small sample sizes for middle school students from Tyrol and from lower secondary school students from Burgenland. Nevertheless, in both cases we have valid data from comparable school types/grades from two other states. Thus, we believe that our conclusions concerning the significant influence of grade, but not of school location are valid. On the positive side answers seem to have been given mostly honestly, without aid from others or the internet, since the points earned were, on average, far below the maximally achievable score.

Concerning our scoring system, scoring may be arguably seen differently for some items. For example, we rated “micro-organisms” as wrong in the definitions what a virus is. As discussed above, even university textbooks often place viruses under this heading. Yet our rating system was guided by the intention to remain as closely as possible to scientific truth also in language.

## Supplementary Material

Supplementary tables and survey.

## Figures and Tables

**Table 1:** Demographic data of study participants

Parameter	Study A		Study B		
	all over Austria		schools in 3 Austrian states		
**Participants**					
Total	1445		2305		
Removed	418		577		
Analyzed	1027		1728		
**Sex (%)**					
Male	39.5		37.3		
Female	58.5		59.7		
Other	1.9		3.0		
**Age (%)**					
below 21	52.9		100		
21 to 40	24.6		/		
41 to 60	17.5		/		
over 60	5.0		/		
**Level of education (%)**					
No final secondary degree	18.1		/		
GCSE	21.9		/		
A-levels	23.8		/		
University degree	25.1		/		
**Residence (%)**					
Burgenland	/		13.7		
Styria	/		67.2		
Tyrol	/		19.2		
**First language (%)**					
German	89.1		83.3		
Others	10.9		16.7		
**Prior knowledge about viruses (%)**					
Yes	16.5		/		
No	83.5		/		
**Interest in viruses (start of survey) (%)**					
Yes	44.9		73.2		
No	55.1		26.8		
**Interest in viruses (end of survey) (%)**					
Yes	/		68.1		
No	/		31.9		
**Grade (%)**					
5^th^	/		2.8		
6^th^	/		4.7		
7^th^	/		5.1		
8^th^	/		18.5		
9^th^	/		20.7		
10^th^	/		15.7		
11^th^	/		10.9		
12^th 1^	/		7.1		
12/13^th 2^	/		13.0		
Others	/		1.3		
**Lower secondary school**	/		31.2		
**Upper secondary school**	/		67.5		
**Others**	/		1.3		
**School type (%)**					
(N)MS	/		20.0		
AHS (lower secondary)	/		11.0		
AHS (upper secondary)			19.3		
BMS	/		1.8		
BORG	/		4.3		
BHAK (economy)	/		12.6		
BHAS	/		3.0		
HTL	/		0.5		
HLW (agriculture)	/		17.1		
PTS	/		4.8		
BAfEP	/		3.0		
HBLA	/		0.5		
Others	/		2.1		
					

^1^ Grad 12 of BHAK, HTL, HLW, BAfEP and HBLA (vocational schools); ^2^ 12 = Final grade of AHS (high school = gymnasium), 13 = final grade of vocational schools providing A-levels and BORG (high school with upper secondary only).

**Table 2:** Classification of viruses by students from grades 8, 10, 12/13 and all grades. For “combination” a cut-off was set at 5 % because of the high number of combinations. “Single answers and in combination” comprises the sums of all participants who had ticked of the respective option either alone or in combination with other options

	Viruses are…																				
	Single answers (in %)								Combinations (in %)						Single answers and in combination (in %)						
Grade	pathogens	microorganisms	a kind of bacteria	unicellular organisms	non-living particles	destroyable by antibiotics	uncertain/do not know	unicellular + pathogens		non-living particles + pathogens	kind of bacteria + pathogens	pathogens + microorganisms	unicellular + pathogens + microorganisms	pathogens		microorganisms	a kind of bacteria	unicellular organisms	non-living particles	destroyable by antibiotics	uncertain/do not know
8^th^	24.7	2.2	5.9	2.8	1.9	1.3	8.1	4.7		1.9	10.3	4.7	4.1	71.6		24.1	32.5	19.1	8.4	12.2	15.0
10^th^	19.1	1.1	1.5	2.9	1.8	0.0	11.4	9.2		7.0	2.9	7.7	8.5	77.2		32.0	12.9	27.6	15.4	9.2	17.3
12/13^th^	18.8	4.0	0.4	1.3	1.8	0.9	7.1	7.6		10.7	4.0	12.9	8.0	80.8		36.2	11.6	24.1	18.3	10.7	13.4
All grades	21.1	2.4	3.6	3.5	2.5	1.1	9.7	6.8		5.6	6.6	7.3	5.9	72.1		27.8	20.4	24.3	14.5	10.3	15.4
																					

**Table 3:** Percentage of participants believing a certain disease to be caused by a virus (viral diseases in bold)

Disease	Percentage classifying this disease as viral (study A)	Percentage classifying this disease as viral (study B)
**Covid-19**	**98**	**91.1**
**Influenza**	**92.9**	**81.7**
**Measles**	**71.3**	**53.5**
**Swine fever**	**57.5**	**49.1**
**Rubella**	**53.1**	**36.1**
**Tick-borne encephalitis** (prominent in many Austrian districts)	**47.8**	**33.4**
	
Malaria	44.4	41.7
The Plague	43.1	51.4
**Cervical cancer** (main cause: human papilloma viruses)	**42.6**	**25**
	
Tuberculosis	23	18.6
Borreliosis	18	16.7

**Table 4:** Students' choices concerning multiplication of viruses. For “combination” a cut-off was set at 5 % because of the high number of combinations. “Single answers and in combination” comprises the sums of all participants who had ticked of the respective option either alone or in combination with other options

	How are viruses multiplied?											
	Single answers (in %)				Combinations (in %)				Single answers and in combination (in %)			
Grade	transfer of genetic material into host cells	through division	by external help	in other viruses	uncertain/do not know	through division + host cell	by external help + host cell	transfer of genetic material into host cells	through division	by external help	in other viruses	uncertain/do not know
8th	9.4	19.4	8.1	12.2	17.8	6.9	8.8	29.4	37.5	23.4	23.4	22.5
10th	11.0	15.8	7.4	6.3	15.8	8.8	17.6	45.6	35.3	30.5	18.8	19.1
12/13th	16.5	13.4	4.9	5.8	14.7	5.4	23.7	56.3	25.4	33.9	17.9	16.5
All grades	12.2	15.9	8.6	8.2	18.2	5.9	14.1	39.3	32	29.1	19.5	21.9

**Table 5:** Students' choices as to how the immune system detects viruses

	How does the immune system detect a virus?				
Grade	antibodies	antigens	shape of the virus	anticells	uncertain/do not know
8^th^	37.2 %	12.8 %	10.3 %	11.6 %	28.1 %
10^th^	30.9 %	27.6 %	11.0 %	7.7 %	22.8 %
12/13^th^	27.2 %	33.5 %	13.8 %	8.5 %	17.0 %
All grades	32.8 %	21.7 %	11.5 %	8.6 %	25.5 %

**Table 6:** Students' choices concerning various correct and incorrect representations of a virus. For “combination” a cut-off was set at 5% because of the high number of combinations. “Single answers and in combination” comprises the sums of all participants who had ticked of the respective option either alone or in combination with other options

	Which of the following pictures show viruses?													
	Single answers (in %)						Combinations (in %)		Single answers and in combination (in %)					
Grade	bacterium	enveloped virus	bacteriophage	animal cell	plant cell	uncertain/do not know	bacteriophage + enveloped virus	bacterium + enveloped virus	bacterium	enveloped virus	bacteriophage	animal cell	plant cell	uncertain/ do not know
8^th^	46.3	9.7	2.8	4.1	2.5	5.3	3.8	6.9	67.5	27.2	10.3	16.3	8.8	8.4
10^th^	30.5	23.5	5.1	5.5	4.0	7.0	8.5	4.0	42.3	38.6	15.8	12.9	9.9	8.5
12/13^th^	29.9	18.3	8.0	4.0	4.0	6.7	14.3	3.1	43.3	39.7	27.2	9.4	10.3	8.9
All grades	35.0	16.0	5.2	4.9	3.4	6.9	7.2	4.3	52.0	33.0	16.9	14.1	10.1	10.7

**Table 7:** Total knowledge score of study A in relation to specific demographic parameters and attitudes

Parameter/Attitude	Achieved points		Post-hoc-test (GT2 Hochberg or Games-Howell) / t-test				Test of normal distribution	
	M	SD	p				Kolmogorov-Smirnov (p)	Shapiro-Wilk (p)
**Sex**								
Male	9.87	2.97	*Reference*	.008	< .001		.006	.017
Female	9.3	2.97	.008	*Reference*	< .001		< .001	< .001
Diverse	6.9	1.94	< .001	< .001	*Reference*		.200	.753
**Age**								
below 21	8.04	2.35	*Reference*	< .001	< .001	< .001	.001	< .001
21 to 40	11.47	2.83	< .001	*Reference*	.011	.410	< .001	.002
41 to 60	10.66	2.59	< .001	.011	*Reference*	.997	.200	.223
over 60	10.75	3.04	< .001	.410	.997	*Reference*	.200	.757
**Level of education**								
No final secondary degree	7.57	2.12	*Reference*	.763	< .001	< .001	.030	.002
GCSE	7.88	2.2	.763	*Reference*	< .001	< .001	.200	.397
A-levels	10.30	2.82	< .001	< .001	*Reference*	< .001	.200	.211
University degree	11.77	2.64	< .001	< .001	< .001	*Reference*	.008	.013
**First Language**								
German	9.75	2.91	*Reference*	< .001			< .001	< .001
Others	7.22	2.59	< .001	*Reference*			.001	< .001
**Prior knowledge about viruses**								
Yes	12.25	2.99	*Reference*	< .001			< .001	< .001
No	8.93	2.66	< .001	*Reference*			.002	.001
**Interest in viruses**								
Yes	10.09	3.07	*Reference*	< .001			.001	< .001
No	8.98	2.82	< .001	*Reference*			.002	< .001
**Willingness to become vaccinated against COVID**								
Strong Yes	10.21	3.11	*Reference*	< .001			.200	.226
Strong No	8.48	2.58	< .001	*Reference*			.079	.164
**Attitude towards mandatory vaccination against COVID**								
Strong Yes	9.03	2.83	*Reference*	.248			.177	.112
Strong No	9.39	2.85	.248	*Reference*			.001	.031
Total	9.48	2.99						
	**Factors of t-test / analyses of variance**							
	**Significance of Levene's test**		**Number of degrees of freedom (df)**		**t-test / ANOVA (F / Welch-F)**	**p**	**d / ⴄ^2^**	**95 % CI of d / ⴄ^2^**
**Analyses of variance**								
Sex	.034		2, 54.076		21.648	< .001	.023	.008 - .044
Age	< .001		3, 196.735		120.883	< .001	.269	.224 - .310
Level of education	< .001		6, 162.671		79.769	< .001	.316	.269 - .355
**t-tests**								
First Language	.006		147.589		9.602	< .001	0.877	0.677 - 1.077
Prior knowledge about viruses	.014		223.423		13.433	< .001	1.222	1.049 - 1.395
Interest in viruses	.007		946.969		5.988	< .001	0.379	0.255 - 0.503
Willingness to become vaccinated against COVID (Strong Yes - Strong No)	.002		406.931		6.143	< .001	0.597	0.397 - 0.795
Attitude towards mandatory vaccination against COVID (Strong Yes - Strong No)	.710		393		1.158	.248	0.129	0.090 - 0.347

**Table 8:** Total knowledge score of study B in relation to specific demographic parameters

	Achieved points		Result of post-hoc-test (GT2 Hochberg or Games-Howell) / t-test																		Test of normal distribution		
Parameter/Attitude	M	SD	p																		Kolmogorov-Smirnov (p)		Shapiro-Wilk (p)
**Sex**																							
Male	7.24	2.72	**Ref.**	.639		< .001															< .001		< .001
Female	7.12	2.43	.639	**Ref.**		< .001															< .001		< .001
Diverse	5.35	2.24	< .001	< .001		**Ref.**															.200		.072
**First Language**																							
German	7.35	2.52	**Ref.**	< .001																	< .001		< .001
Others	5.94	2.40	< .001	**Ref.**																	.038		< .001
**Grade**																							
5^th^	6.19	2.72	**Ref.**	.888		.999	1		.741	.026	.002		.035	< .001	1						.070		< .001
6^th^	5.49	2.34	.888	**Ref.**		.996	.264		< .001	< .001	< .001		< .001	< .001	1						.200		.900
7^th^	5.82	2.49	.999	.996		**Ref.**	.946		.010	< .001	< .001		< .001	< .001	1						.014		.012
8^th^	6.21	2.09	1	.264		.946	**Ref.**		.002	< .001	< .001		< .001	< .001	.999						.003		< .001
9^th^	6.92	2.46	.741	< .001		.010	.002		**Ref.**	.007	< .001		.084	< .001	.613						< .001		< .001
10^th^	7.66	2.38	.026	< .001		< .001	< .001		.007	**Ref.**	.870		1	.037	.076						.006		.002
11^th^	8.0	2.27	.002	< .001		< .001	< .001		< .001	.870	**Ref.**		.993	.806	.021						.037		.047
12^th^	7.72	2.55	.035	< .001		< .001	< .001		.084	1	.993		**Ref.**	.359	.076						.200		.051
12/13^th^	8.4	2.63	< .001	< .001		< .001	< .001		< .001	.037	.806		.359	**Ref.**	.004						.003		< .001
Others	5.8	2.64	1	1		1	.999		.613	.076	.021		.076	.004	**Ref.**						.200		.769
**School level**																							
Upper sec.	6.04	2.27	**Ref.**	< .001																	< .001		< .001
Lower sec.	7.64	2.51	< .001	**Ref.**																	< .001		< .001
**School type**																							
(N)MS	5.34	1.98	**Ref.**	< .001		< .001	.006		< .001	< .001	< .001		1	< .001	1	< .001	.010		.226		.001		< .001
AHS (lower sec.)	7.36	2.22	< .001	**Ref.**		< .001	1		.273	1	.994		.999	1	< .001	1	.989		.853		< .001		< .001
AHS (upper sec.)	8.78	2.62	< .001	< .001		**Ref.**	.049		.871	< .001	< .001		.807	< .001	< .001	.009	.754		.001		.010		.001
BMS	7.22	2.31	.006	1		.049	**Ref.**		.727	1	1		1	1	.015	1	.995		.955		< .001		< .001
BORG	8.23	2.44	< .001	.273		.871	.727		**Ref.**	.116	.108		.945	.104	< .001	.912	1		.071		.200		.380
BHAK (econ.)	7.23	2.34	< .001	1		< .001	1		.116	**Ref.**	1		1	1	< .001	.993	.955		.947		.015		.006
BHAS	6.96	2.11	< .001	.994		< .001	1		.108	1	**Ref.**		1	.999	.002	.920	.853		1		.200		.875
HTL	6.18	4.07	1	.999		.807	1		.945	1	1		**Ref.**	1	1	.996	.987		1		.055		.213
HLW (agric.)	7.26	2.29	< .001	1		< .001	1		.104	1	.999		1	**Ref.**	< .001	.993	.958		.926		.001		.001
PTS	5.44	1.56	1	< .001		< .001	.015		< .001	< .001	.002		1	< .001	**Ref.**	< .001	.012		.412		.200		.415
BAfEP	7.6	1.85	< .001	1		.009	1		.912	.993	.920		.996	.993	< .001	**Ref.**	1		.659		.200		.858
HBLA	7.88	1.32	.010	.989		.754	.995		1	.955	.853		.987	.958	.012	1	**Ref.**		.614		.200		.563
Others	6.56	2.55	.226	.853		.001	.995		.071	.947	1		1	.926	.412	.659	.614		**Ref.**		.200		.976
**Willingness to become vaccinated against COVID**																							
Strong Yes	8.23	2.78	**Ref.**	< .001																	.200		.305
Strong No	6.22	2.24	< .001	**Ref.**																	.022		.012
**Attitude towards mandatory vaccination against COVID**																							
Strong Yes	7.21	2.41	**Ref.**	.107																	.200		.383
Strong No	6.86	2.54	.107	**Ref.**																	< .001		< .001
Total	7.11	2.55																					
	**Factors of t-test / analyses of variance**																						
**Parameter/Attitude**					**Significance of Levene's test**			**Number of degrees of freedom (df)**				**t-test / ANOVA (F / Welch-F)**						**p**		**d / ⴄ^2^**		**95 % CI of d / ⴄ^2^**	
**Analyses of variance**																							
Sex					.002			2, 140.474				16.672						< .001		.015		.005 - .028	
Grade					.028			9, 307.673				26.065						< .001		.121		.090 - .146	
School type					< .001			12, 141.036				40.837						< .001		.213		.176 - .241	
State (in combination with lower and upper secondary school)					.002			4, 1719				2.461						.044*		.006			
State (in combination with lower and upper secondary school as well as school type)					< .001			3, 1680				2.131						.094		.004			
**t-tests**																							
First Language					.270			1726				8.724						< .001		0.562		0.435 - 0.690	
Secondary school level (lower - upper)					.007			1148.701				13.089						< .001		0.657		0.553 - 0.761	
Willingness to become vaccinated against COVID (Strong Yes - Strong No)					< .001			318.556				9.212						< .001		0.832		0.665 - 1.0	
Attitude towards mandatory vaccination against COVID (Strong Yes - Strong No)					.723			776				1.612						.107		0.139		-0.30 - 0.307	
																							

* No significance because significance threshold was lowered to .01 due to inhomogeneity of variance.
