# Supplementary material for: Virus-related Knowledge in Covid-19 Times - Results from two Cross-sectional Studies in Austria and Implications for School
Source: Int J Biol Sci. 2022 Jan 31;18(4):1627–50. doi: 10.7150/ijbs.69224 (PMC8898374; doi:10.7150/ijbs.69224)
Supplement: Supplementary file 1 — Supplementary tables and survey. [file ijbsv18p1627s1.pdf]

Table S1

A. Retest-reliability for study A (Pearson's r and intraclass-correlation). Values are given for the three knowledge domains tested, and for the total questionnaire.

Retest-reliability			
Domain	*R*	*ICC*	95%-CI (ICC)
Coronavirus	.624***	.624***	.405 - .776
Vaccination	.735***	.733***	.560 - .845
Viruses (general)	.848***	.842***	.729 - .911
Total	.892***	.892***	.797 - .935

*** $p \leq .001$

B. Retest-reliability for study B (Pearson's r and intraclass-correlation). Values are given for the three knowledge domains tested, and for the total questionnaire.

Retest-reliability			
Domain	*R*	*ICC*	95%-CI (ICC)
Coronavirus	.694***	.692***	.539 - .801
Vaccination	.659***	.657***	.492 - .777
Viruses (general)	.627***	.625***	.449 - .754
Total	.790***	.787***	.672 - .865

*** $p \leq .001$

Survey S1

Survey About Knowledge Related to Viruses – Student Version

Dear Student!

Corona influences our life since spring last year – and that also holds true for school. Yet how good do YOU feel informed about this virus and viruses in general? What would YOU wish to learn at school and find in schoolbooks? The University of Graz has developed a survey, with various questions where you can test your knowledge but also note down your opinion. This survey is completely anonymous. It takes about 10-15 minutes and you can stop it whenever you want. It is in accordance with all data protection regulations.

It is important that you answer each question honestly and without any aid. Your answers may contribute to the decision, whether some and, if so, which topics should be dealt with more intensely or even newly at school and in schoolbooks.

We are grateful that you take the time to participate in this survey and are excited to read you answers!

Prof. Dr. Uwe Simon & Marc Bracko, BEd

One further remark concerning personal data protection: This is an anonymous survey. This means that it is impossible to identify any participant, even though some personal data are asked for (e.g., grade). The University of Graz takes data protection regulations very seriously and treats any person-related data confidential and according to law. Thus, we are unable to identify, who ticked off a particular answer.

How interesting is the topic "viruses" for you?

[Please, choose one answer only.]

- Highly interesting
- Interesting
- Uninteresting
- Absolutely uninteresting

During the pandemic, much has been speculated about the origin of SARS-CoV-2. Which of the following statements comes closest to what YOU think?

[Please, choose one answer only.]

- **The virus was transferred from animals to humans in a natural way.**
- The virus has been developed intentionally by humans in a lab.
- The virus actually does not exist.
- Don't know / uncertain.

Which is the correct name of the virus which we call "coronavirus" in everyday language?

[Please, choose one answer only.]

- Covid-19
- **SARS-CoV-2**
- MERS-CoV-2
- SARS-CoV-1
- Don't know / uncertain

How can the coronavirus be transmitted between humans?

[Please, choose all answers you believe are correct.]

- **Droplets**
- **Aerosols**
- Animal bites
- Food
- **Contact with contaminated surfaces**
- Don't know / uncertain

During the whole pandemic the role of children in spreading the virus was discussed repeatedly. Do children play a role in transmission of the coronavirus?

[Please, choose all answers you believe are correct.]

- **Yes: Infected children may spread the virus to same-age children.**
- **Yes: Infected children may spread the virus to adults.**
- No: The virus load in the throat of infected children is too low for transmitting it to others.
- Don't know / uncertain.

Which of the following is usually done to find out whether an individual has a coronavirus infection?

[Please, choose all answers you believe are correct.]

- **Nasal swab**
- Blood sampling
- **Throat swab**
- Urine sampling
- Don't know / uncertain

Which of the following measure do you think is most effective to slow down the coronavirus pandemic?

[Please, choose all answers you believe are correct.]

- Obligatory mask wearing in rooms
- Regular hand washing
- Keeping distance to others
- Lockdown (closing down schools, shops, etc.)
- Contact tracing
- Others[1]

[1] please specify:

Presently, at least 266.875 died in the U.S. with or through the coronavirus (30.11.2020). Please estimate, how many people died due to flue (influenza) in the U.S. in the season 2018/19.

[Please, choose one answer only.]

- **Less than 50.001** [note for readers: exact number was 34.200 (CDC, 2020)]
- 50.001 to 150.000
- 150.001 to 300.000
- 300.001 to 500.000
- More than 500.000

Please estimate, how many people died due to flue (influenza) in Austria in the season 2018/19.

[Please, choose one answer only.]

- Less than 501
- 501 to 1,000
- **1,001 to 1,500** [note for readers: exact number was 1.373 (AGES, 2020)]
- 1,501 to 2,000
- 2,001 to 2,500
- More than 2,500

If there were a vaccine against the coronavirus available tomorrow, would you take it?

[Please, choose one answer only.]

- Strong Yes
- Rather Yes
- Don’t know / uncertain
- Rather No
- Strong No

Please, explain your answer:

If there were a vaccine against the coronavirus available, should vaccination become obligatory by law?

[Please, choose one answer only.]

- Strong Yes
- Rather Yes
- Don't know / uncertain
- Rather No
- Strong No

Against which of the following does vaccination partly offer protection?

[Please, choose all answers you believe are correct.]

- **Bacteria**
- **Viruses**
- Fungi
- Don't know / uncertain

Which of the following can a vaccine contain?

[Please, choose all answers you believe are correct.]

- **Attenuated pathogens**
- **Antibodies against pathogens**
- **Inactivated pathogens**
- Antibiotics against pathogens
- Don't know / uncertain

A person had received the influenza vaccination last year. Yet she has fallen ill due to influenza now. What reasons could this have had?

[Please, choose all answers you believe are correct.]

- **Viruses may mutate and thereby change their characteristics.**
- This is not possible, since vaccinations provide a 100% protection against a disease.
- The injected vaccine dose in the previous year was too small.
- **Vaccination may not work in individual cases.**
- Don't know / uncertain

Sometimes, vaccinations have side-effects or even vaccination damage. Vaccination damage means that a person suffers from lasting damage after a correctly delivered vaccination. Please estimate how many cases of vaccination damage occurred in Austria between 1990 and 2019 (within the last 30 years).

[Please, choose one answer only.]

- Less than 101
- **101 to 1,000** [note for readers: exact number was 409 (BMSGPK, 2020)]
- 1,001 to 2,000
- 2,001 to 3,000
- 3,001 to 4,000
- 4,001 to 5,000
- More than 5,000

When speaking about communicable diseases, a term often discussed is "herd immunity". Herd immunity is reached, when the spread of a specific disease within a population is almost stopped, because a specific percentage of this population is already immune against the pathogen which causes this disease. Which percentage of the population has to be immune against measles to reach herd immunity against this disease?

[Please, choose one answer only.]

- Approx. 55 %
- Approx. 65 %
- Approx. 75 %
- Approx. 85 %
- **Approx. 95 %**

Viruses are ...

[Please, choose all answers you believe are correct.]

- Unicellular organisms
- **Non-living particles**
- Destroyable with antibiotics
- A kind of bacteria
- **Pathogens**
- Microorganisms
- Don't know / uncertain

Which of the following statements about viruses and bacteria are correct?

[Please, choose all answers you believe are correct.]

- Bacteria are smaller than viruses.
- **Bacteria are more complex than viruses** (e.g., contain organelles).
- Antibiotics are efficient against viruses and bacteria.
- Don't know / uncertain

Where can you find viruses?

[Please, choose all answers you believe are correct.]

- **In humans**
- **In animals**
- **In plants**
- **In bacteria**
- **In fungi**
- Don't know / uncertain

Which of the following diseases are caused by viruses?

Disease	Yes	No	Don't know/uncertain
**Measles**			
Tuberculosis			
The Plague			
Borreliosis			
**Tick-borne encephalitis**			
**Rubella**			
**Cervical cancer**			
**Covid-19**			
**Swine fever**			
**Influenza**			
Malaria			

How do viruses multiply?

[Please, choose all answers you believe are correct.]

- By division, so that one virus splits into two new viruses
- **With external aid, because they don't have a metabolism on their own**
- By delivering their genetic material to other viruses
- **By injecting their genetic material into their host cells**
- Don't know / uncertain

Whereby does the human immune system recognize a virus?

[Please, choose one answer only.]

- By the antibodies of the virus
- **By the antigens of the virus**
- By the anticells of the virus
- Due to the form of the virus
- Don't know / uncertain

Which of the following pictures represent a virus?

[Please, choose all answers you believe are correct.]

1)

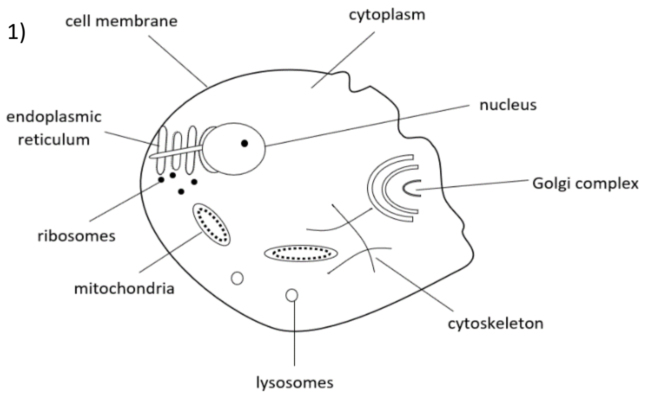

2)

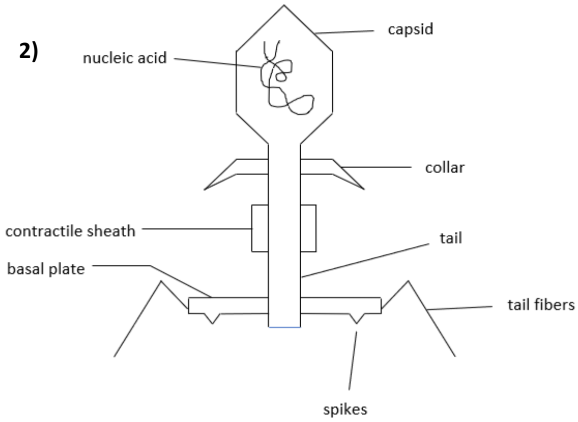

3)

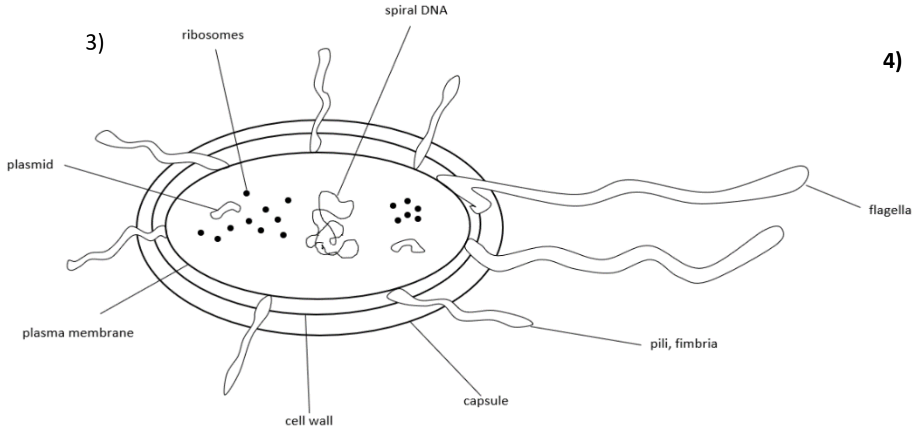

4)

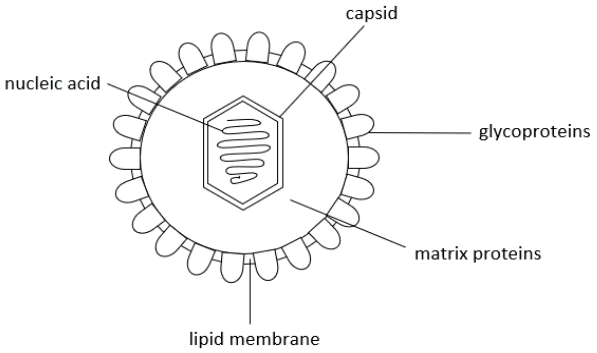

5)

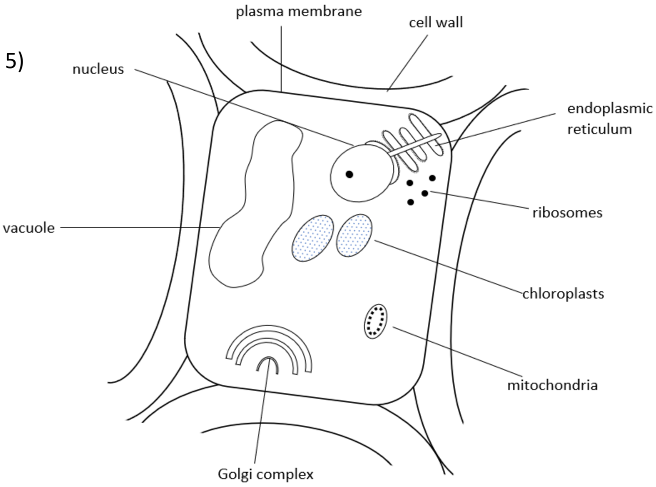

How well-informed do you feel about viruses by what you have learnt at school?

[Please, choose one answer only.]

- Very well
- Well
- Moderately[1]
- Badly[1]
- Very badly[1]

[1]About which topic would you like to be provided with more information?

How exiting is the topic "virus" for you?

[Please, choose one answer only.]

- Very exciting
- Exciting
- Boring
- Very boring

Demographic data

Participation in this survey is voluntary. All information is kept strictly confidential. No answer you give can be tracked down to an individual. However, for this research we would be grateful for the following information:

Gender:

[Please, choose one answer only.]

- Male
- Female
- Other
- No answer

In which district do you live?

[Please, choose one answer only.]

[List of districts for each participating state to choose from.]

German is your …

[Please, choose one answer only.]

- First language
- Second language[1]
- No answer

[1]What is your first language?

Which grade do you attend?

[Please, choose one answer only.]

[List of grades to choose from.]

Which type of school do you attend?

[Please, choose one answer only.]

[List of school types to choose from.]

Table S2. Scoring System

+ 0.5 points for each correct answer (both single and multiple choice)

- 0.5 points for each incorrect answer (multiple choice)

No reduction for “don’t know/uncertain”

No reduction below zero (zero points as baseline for each item).

+/- 0.25 points for each correct/incorrect answer for item “diseases” to not overweigh this item

Item	Number of correct choices	Possible score
**Domain “Coronavirus“**		
During the pandemic, much has been speculated about the origin of SARS-CoV-2. Which of the following statements comes closest to what YOU think?	1	0.5
Which is the correct name of the virus which we call “coronavirus” in everyday language?	1	0.5
How can the coronavirus be transmitted between humans?	3	1.5
During the whole pandemic the role of children in spreading the virus was discussed repeatedly. Do children play a role in transmission of the coronavirus?	2	1
Which of the following is usually done to find out whether an individual has an coronavirus infection?	2	1
Presently, at least 266.875 died in the U.S. with or through the coronavirus (30.11.2020). Please estimate, how many people died due to flue (influenza) in the U.S. in the season 2018/19.	1	0.5
Please estimate, how many people died due to flue (influenza) in Austria in the season 2018/19.	1	0.5
**Total (Coronavirus):**	**11**	**5.5**

**Domain "Vaccination"**		
Against which of the following does vaccination partly offer protection?	2	1
Which of the following can a vaccine contain?	3	1.5
A person had received the influenza vaccination last year. Yet she has fallen ill due to influenza now. What reasons could this have had?	2	1
Sometimes, vaccinations have side-effects or even vaccination damage. Vaccination damage means that a person suffers from lasting damage after a correctly delivered vaccination. Please estimate how many cases of vaccination damage occurred in Austria between 1990 and 2019 (within the last 30 years).	1	0.5
When speaking about communicable diseases, a term often discussed is "herd immunity". Herd immunity is reached, when the spread of a specific disease within a population is almost stopped, because a specific percentage of this population is already immune against the pathogen which causes this disease. Which percentage of the population has to be immune against measles to reach herd immunity against this disease?	1	0.5
**Total (Vaccination):**	**9**	**4.5**
**Domain "Viruses"**		
Viruses are ...	2	1
Which of the following statements about viruses and bacteria are correct?	1	0.5
Where can you find viruses?	5	2
Which of the following diseases are caused by viruses?		
• *Measles*	1	0.25
• *Tuberculosis*	1	0.25
• *The Plague*	1	0.25
• *Borreliosis*	1	0.25

• *Tick-borne encephalitis*	1	0.25
• *Rubella*	1	0.25
• *Cervical cancer*	1	0.25
• *Covid-19*	1	0.25
• *Swine fever*	1	0.25
• *Influenza*	1	0.25
• *Malaria*	1	0.25
How do viruses multiply?	2	1
Whereby does the human immune system recognize a virus?	1	0.5
Which of the following pictures represent a virus?	2	1
**Total (Viruses):**	**24**	**8.75**
**Total (complete survey)**	**44**	**18.75**

Table S3. Knowledge scores for the domain coronavirus of study A in relation to specific demographic parameters.

Parameter	Achieved points		Post-hoc-test (GT2 Hochberg or Games-Howell) / t-test				Test of normal distribution	
	M	SD	p				Kolmogorov-Smirnov (p)	Shapiro-Wilk (p)
**Sex**								
Male	3.52	0.92	*Reference*	.025	.016		< .001	< .001
Female	3.37	0.88	.025	*Reference*	.113		< .001	< .001
Diverse	2.95	1.09	.016	.113	*Reference*		.048	.251
**Age**								
below 21	3.12	0.89	*Reference*	< .001	< .001	.001	< .001	< .001
21 to 40	3.85	0.75	< .001	*Reference*	.166	.218	< .001	< .001
41 to 60	3.68	0.82	< .001	.166	*Reference*	.987	< .001	< .001
over 60	3.59	0.84	.001	.218	.987	*Reference*	< .001	.008
**Level of education**								
No final secondary degree	3.04	0.82	*Reference*	1	< .001	< .001	< .001	< .001
GCSE	3.09	0.92	1	*Reference*	< .001	< .001	< .001	< .001
A-levels	3.65	0.81	< .001	< .001	*Reference*	.131	< .001	< .001
University degree	3.86	0.8	< .001	< .001	.131	*Reference*	< .001	< .001
**First Language**								
German	3.50	0.87	*Reference*	< .001			< .001	< .001
Others	2.79	0.93	< .001	*Reference*			< .001	.019
**Prior knowledge about viruses**								
Yes	3.70	0.85	*Reference*	< .001			< .001	< .001
No	3.37	0.90	< .001	*Reference*			< .001	< .001
**Interest in viruses**								
Yes	3.48	0.87	*Reference*	.061			< .001	< .001
No	3.38	0.92	.061	*Reference*			< .001	< .001
Total	3.42	0.90						

Parameter	Factors of t-test / analyses of variance					
	Significance of Levene´s test	Number of degrees of freedom (df)	t-test / ANOVA (F / Welch-F)	p	d / η^2	95 % CI of d / η^2
			Analyses of variance			
Sex	.273	2, 1024	6.329	.002	.012	.002 – .028
Age	.097	3, 1023	50.973	< .001	.130	.093 – .166
Level of education	.238	6, 1020	28.648	< .001	.144	.103 – .179
			t-tests			
First Language	.309	1025	8.113	< .001	0.812	0.613 – 1.011
Prior knowledge about viruses	.534	1025	4.474	< .001	0.376	0.211 – 0.542
Interest in viruses	.191	1025	1.878	.061	0.118	−0.005 – 0.241

Summary:

Males scored significantly more points than females. The knowledge difference was greatest between those below 21 and those between 21 and 40. With respect to education, those with a university degree performed significantly better than participants with GCSE as highest degree and participants without final secondary school grades, but not significantly better than A-levels. Participants with A-levels also earned significantly more points than participants with GCSE and without final secondary degree. Moreover, people who regarded themselves as equipped with some virology knowledge performed significantly better than those who did not, and participants with German as mother tongue also reached significantly more points than those with other languages.

Table S4. Knowledge scores for the domain coronavirus of study B in relation to specific demographic parameters.

	Achieved points		Result of post-hoc-test (GT2 Hochberg or Games-Howell) / t-test										Test of normal distribution	
Parameter	M	SD	p										Kolmogorov-Smirnov (p)	Shapiro-Wilk (p)
Sex														
Male	2.83	1.02	**Ref**.	.995	< .001								< .001	< .001
Female	2.82	0.93	.995	**Ref.**	< .001								< .001	< .001
Diverse	2.14	0.97	< .001	< .001	**Ref.**								< .001	.012
First Language														
German	2.89	0.94	**Ref.**	< .001									< .001	< .001
Others	2.38	1.02	< .001	**Ref.**									< .001	< .001
Grade														
5th	2.57	0.89	**Ref.**	.820	.991	1	.989	.145	.064	.588	.001	.842	.012	.022
6th	2.3	0.96	.820	**Ref.**	.999	.143	.022	< .001	< .001	.001	< .001	1	.009	.064
7th	2.41	0.92	.991	.999	**Ref.**	.605	.178	< .001	< .001	.012	< .001	.992	.002	.018
8th	2.63	0.97	1	.143	.605	**Ref.**	.989	.001	< .001	.291	< .001	.526	< .001	< .001
9th	2.71	1.03	.989	.022	.178	.989	**Ref.**	.038	.012	.819	< .001	.304	< .001	< .001
10th	2.96	0.89	.145	< .001	< .001	.001	.038	**Ref.**	.999	.997	.062	.027	< .001	< .001
11th	3.03	0.92	.064	< .001	< .001	< .001	.012	.999	**Ref**.	.929	.576	.014	< .001	< .001
12th	2.88	0.92	.588	.001	.012	.291	.819	.997	.929	**Ref.**	.042	.084	< .001	.002

12/13th	3.20	0.83	.001	< .001	<.001	< .001	< .001	.062	.576	.042	**Ref.**	.002				< .001	< .001
Others	2.2	0.95	.842	1	.992	.526	.304	.027	.014	.084	.002	**Ref.**				.144	.273
School level																	
Upper secondary	2.54	0.96	**Ref.**	< .001												< .001	< .001
Lower secondary	2.93	0.95	< .001	**Ref.**												< .001	< .001
School type																	
(N)MS	2.29	0.9	**Ref.**	< .001	< .001	.161	< .001	< .001	.017	1	< .001	.503	.007	.216	1	< .001	< .001
AHS (lower secondary)	2.99	0.91	< .001	**Ref.**	.184	.980	1	1	.999	.982	.999	< .001	1	.967	.125	< .001	< .001
AHS (upper secondary)	3.23	0.92	< .001	.184	**Ref.**	.229	.557	.011	.296	.879	.001	< .001	.327	.289	.003	< .001	< .001
BMS	2.77	0.85	.161	.980	.229	**Ref.**	.995	.999	1	1	1	.009	1	1	.949	.043	.080
BORG	2.98	0.85	< .001	1	.557	.995	**Ref.**	1	1	.986	1	< .001	1	.991	.222	.001	.001
BHAK (economy)	2.92	0.92	< .001	1	.011	.999	1	**Ref.**	1	.992	1	< .001	1	.998	.264	< .001	< .001
BHAS	2.84	0.97	.017	.999	.296	1	1	1	**Ref.**	.998	1	< .001	1	1	.788	.034	.124
HTL	2.38	1.48	1	.982	.879	1	.986	.992	.998	**Ref.**	.994	1	.998	1	1	.200	.492
HLW (agriculture)	2.91	0.89	< .001	.999	.001	1	1	1	1	.994	**Ref.**	< .001	1	.999	.282	< .001	< .001
PTS	2.05	0.83	.503	< .001	< .001	.009	< .001	< .001	< .001	1	< .001	**Ref.**	< .001	.029	.740	< .001	.007
BAfEP	2.86	0.93	.007	1	.327	1	1	1	1	.998	1	< .001	**Ref.**	1	.715	.001	.016
HBLA	2.78	0.44	.216	.967	.289	1	.991	.998	1	1	.999	.029	1	**Ref.**	.933	.116	.338
Others	2.43	1.02	1	.125	.003	.949	.222	.264	.788	1	.282	.740	.715	.993	**Ref.**	.008	.063

Total	2.80	0.97

Parameter	Factors of t-test / analyses of variance					
	Significance of Levene´s test	Number of degrees of freedom (df)	t-test / ANOVA (F / Welch-F)	p	d / η^2	95 % CI of d / η^2
			Analyses of variance			
Sex	.004	2, 138.240	12.586	< .001	.015	.005 – .027
Grade	.012	9, 309.964	14.863	< .001	.068	.043 – .087
School type	.033	12, 141.432	21.875	< .001	.135	.101 – .159
State (in combination with upper and lower secondary school)	.596	4, 1719	2.538	.038	.006	
State (in combination with upper and lower secondary as well as school type)	.034	3, 1680	1.011	.387	.002	
			t-tests			
First Language	.105	1726	8.304	< .001	0.535	0.408 – 0.663
Secondary school level (lower – upper)	.696	1703	7.959	< .001	0.415	0.311 – 0.518

Summary:

There was no significant difference between females and males. Comparing lower and upper secondary students separately across the three participating states showed no significant influence of school location. On the other hand, knowledge scores were significantly different between lower and upper secondary students as such: Upper secondary students gained in average 2.93 points, those from lower secondary 2.54 (d = 0.415). In upper secondary school, students of grade 12/13 (last year of upper secondary school) gained significantly more points than students from grade 8 (last year of lower secondary) as did students from grade 10. No significant difference was observed between grades 12/13 and grade 10. Second, ANOVA revealed a highly significant group effect for school type (Welch-F(12, 141.432) = 21.875, p < .001, η2 = .135, 95% - CI for η2 [.101, .159]): For example, students from lower secondary high school gained significantly more points than students from middle school. Significant differences were also observed between students with German as first language and those with other first languages. Comparing lower and upper secondary students of same school types across the three participating states showed no significant differences (F(3, 1680) = 1.011, p = .387, η2 = .002). Tables 1-3 show how each subgroup scored for each domain and in total.

Table 1. Average knowledge scores for study B – comparison of students from middle and from general high school (gymnasium; most often attended high school type in Austria).

	Participants (n)			Domain coronavirus			Domain vaccination			Domain viruses in general			Total knowledge		
General School type	Burgen-land	Styria	Tyrol	Burgen-land	Styria	Tyrol	Burgen-land	Styria	Tyrol	Burgen-land	Styria	Tyrol	Burgen-land	Styria	Tyrol
Middle school (grades 5-8)	46	284	16	2.52 (SD = 0.86)	2.25 (SD = 0.87)	2.31 (SD = 1.38)	1.6 (SD = 0.7)	1.3 (SD = 0.75)	1.38 (SD = 1.2)	1.94 (SD = 0.78)	1.64 (SD = 0.91)	2.25 (SD = 1.77)	6.05 (SD = 1.64)	5.18 (SD = 1.86)	5.94 (SD = 3.86)
General high school, lower secondary (grades 5-8)	3	143	47	2.83 (SD = 0.29)	2.97 (SD = 0.96)	3.07 (SD = 0.77)	2.33 (SD = 0.29)	1.94 (SD = 0.77)	1.94 (SD = 0.83)	1.67 (SD = 1.54)	2.35 (SD = 1.23)	2.54 (SD = 1.32)	6.84 (SD = 1.53)	7.26 (SD = 0.77)	7.55 (SD = 2.35)
General high school, upper secondary (grades 9-12)	40	179	115	2.84 (SD = 0.93)	3.31 (SD = 0.93)	3.22 (SD = 0.86)	1.89 (SD = 0.73)	2.36 (SD = 0.81)	2.37 (SD = 0.78)	2.7 (SD = 1.38)	3.24 (SD = 1.72)	3.43 (SD = 1.47)	7.42 (SD = 2.10)	8.92 (SD = 0.81)	9.02 (SD = 2.30)

Table 2. Average knowledge scores for study B – comparison of students from middle and from all types of high school.

School type	Participants (n)			Domain coronavirus			Domain vaccination			Domain viruses in general			Total knowledge		
	Burgen-land	Styria	Tyrol	Burgen-land	Styria	Tyrol	Burgen-land	Styria	Tyrol	Burgen-land	Styria	Tyrol	Burgen-land	Styria	Tyrol
Middle school (grades 5-8)	46	284	16	2.52 (SD = 0.86)	2.25 (SD = 0.87)	2.31 (SD = 1.38)	1.6 (SD = 0.7)	1.3 (SD = 0.75)	1.38 (SD = 1.2)	1.94 (SD = 0.78)	1.64 (SD = 0.91)	2.25 (SD = 1.77)	6.05 (SD = 1.64)	5.18 (SD = 1.86)	5.94 (SD = 3.86)
High school, lower secondary (grades 5-8)	3	143	47	2.83 (SD = 0.29)	2.97 (SD = 0.96)	3.07 (SD = 0.77)	2.33 (SD = 0.29)	1.94 (SD = 0.77)	1.94 (SD = 0.83)	1.67 (SD = 1.54)	2.35 (SD = 1.23)	2.54 (SD = 1.32)	6.84 (SD = 1.53)	7.26 (SD = 0.77)	7.55 (SD = 2.35)
Upper secondary (grades 9-12/13)[1]	184	721	261	2.68 (SD = 0.98)	2.98 (SD = 0.95)	2.99 (SD = 0.89)	1.97 (SD = 0.83)	2.03 (SD = 0.86)	2.05 (SD = 0.83)	2.55 (SD = 1.33)	2.65 (SD = 1.47)	2.84 (SD = 1.47)	7.20 (SD = 2.37)	7.66 (SD = 2.56)	7.89 (SD = 2.44)
Others[2]	3	13	7	2.00 (SD = 0.5)	2.11 (SD = 1.10)	2.43 (SD = 0.84)	1.17 (SD = 1.04)	1.42 (SD = 0.93)	1.92 (SD = 1.21)	2.2 (SD = 0.43)	1.97 (SD = 1.23)	2.16 (SD = 2.31)	5.37 (SD = 1.70)	5.51 (SD = 2.55)	6.52 (SD = 3.27)

[1] Most high school types end with grade 12, some with grade 13. High school types other than the general high school only offer upper secondary school.
[2] Participants who opted for „other“, but did not specify their school type or wrote something incomprehensible.

Table 3. Average knowledge scores for study B – comparison of students from lower and from upper secondary grades.

School level	Participants (n)			Domain coronavirus			Domain vaccination			Domain viruses in general			Total knowledge		
	Burgen-land	Styria	Tyrol	Burgen-land	Styria	Tyrol	Burgen-land	Styria	Tyrol	Burgen-land	Styria	Tyrol	Burgen-land	Styria	Tyrol
Lower secondary (grades 5-8)	49	427	63	2.54 (SD = 0.84)	2.49 (SD = 0.96)	2.88 (SD = 1.00)	1.64 (SD = 0.7)	1.50 (SD = 0.81)	1.79 (SD = 0.96)	1.92 (SD = 0.23)	1.87 (SD = 1.08)	2.47 (SD = 1.44)	6.10 (SD = 1.63)	5.86 (SD = 2.19)	7.14 (SD = 2.86)
Upper secondary (grades 9-12/13)	184	721	261	2.68 (SD = 0.98)	2.98 (SD = 0.95)	2.99 (SD = 0.89)	1.97 (SD = 0.83)	2.03 (SD = 0.86)	2.05 (SD = 0.83)	2.55 (SD = 1.33)	2.65 (SD = 1.47)	2.84 (SD = 1.47)	7.20 (SD = 2.37)	7.66 (SD = 2.56)	7.89 (SD = 2.44)
Others	3	13	7	2.00 (SD = 0.5)	2.11 (SD = 1.10)	2.43 (SD = 0.84)	1.17 (SD = 1.04)	1.42 (SD = 0.93)	1.92 (SD = 1.21)	2.2 (SD = 0.43)	1.97 (SD = 1.23)	2.16 (SD = 2.31)	5.37 (SD = 1.70)	5.51 (SD = 2.55)	6.52 (SD = 3.27)

Table S5. Knowledge score for the domain vaccination of study A in relation to specific demographic parameters.

	Achieved points		Post-hoc-test (GT2 Hochberg or Games-Howell) / t-test				Test of normal distribution	
Parameter	M	SD	p				Kolmogorov-Smirnov (p)	Shapiro-Wilk (p)
				Sex				
Male	2.54	0.83	*Reference*	.043	.002		< .001	< .001
Female	2.40	0.87	.043	*Reference*	.019		< .001	< .001
Diverse	1.88	0.81	.002	.019	*Reference*		.060	.144
				Age				
below 21	2.08	0.81	*Reference*	< .001	< .001	< .001	< .001	< .001
21 to 40	2.85	0.72	< .001	*Reference*	1	.944	< .001	< .001
41 to 60	2.84	0.72	< .001	1	*Reference*	.930	< .001	< .001
over 60	2.91	0.7	< .001	.944	.930	Reference	< .001	.004
				Level of education				
No final secondary degree	1.87	0.75	*Reference*	.020	< .001	< .001	< .001	< .001
GCSE	2.12	0.8	.020	*Reference*	< .001	< .001	< .001	< .001
A-levels	2.69	0.77	< .001	< .001	*Reference*	< .001	< .001	< .001
University degree	2.98	0.64	< .001	< .001	< .001	*Reference*	< .001	< .001
				First Language				
German	2.53	0.82	*Reference*	< .001			< .001	< .001
Others	1.73	0.78	< .001	*Reference*			< .001	< .001
				Prior knowledge about viruses				
Yes	2.93	0.78	*Reference*	< .001			< .001	< .001
No	2.35	0.84	< .001	*Reference*			< .001	< .001
				Interest in viruses				
Yes	2.56	0.86	*Reference*	< .001			< .001	< .001
No	2.36	0.85	< .001	*Reference*			< .001	< .001
Total	2.45	0.86						

	Factors of t-test / analyses of variance					
Parameter	Significance of Levene´s test	Number of degrees of freedom (df)	t-test / ANOVA (F / Welch-F)	p	d / η^2	95 % CI of d / η^2
			Analyses of variance			
Sex	.451	2, 1024	7.606	.001	.015	.003 – .031
Age	.025	3, 209.058	87.789	< .001	.203	.160 – .243
Level of education	.002	6, 159.880	56.955	< .001	.240	.193 – .278
			t-tests			
First Language	.623	1025	9.781	< .001	0.979	0.778 – 1.180
Prior knowledge about viruses	.028	251.910	8.775	< .001	0.700	0.532 – 0.868
Interest in viruses	.888	1025	3.832	< .001	0.240	0.117 – 0.364

Summary:

Males earned significantly more points than females. Participants above 60 yrs. performed best. The knowledge difference was greatest between those below 21 and those over 60. Participants between 21 and 40 as well as between 41 and 60 also scored significantly more points than participants below 21. With respect to education, those with a university degree performed significantly better than all other groups. Participants with A-levels also got significantly more points than participants with GCSE and without final secondary degree. Furthermore, people with GCSE scored significantly more points than people without final secondary degree. People who regarded themselves as equipped with some virology knowledge performed significantly better than those who did not. Participants with German as mother tongue also reached significantly more points than those with other languages.

Table S6. Knowledge score for the domain vaccination of study B in relation to specific demographic parameters.

	Achieved points		Result of post-hoc-test (GT2 Hochberg or Games-Howell) / t-test										Test of normal distribution	
Parameter	M	SD	p										Kolmogorov-Smirnov (p)	Shapiro-Wilk (p)
Sex														
Male	1.87	0.89	**Ref.**	.789	< .001								< .001	< .001
Female	1.90	0.85	.789	**Ref.**	< .001								< .001	< .001
Diverse	1.36	0.82	< .001	< .001	**Ref.**								.001	.011
First Language														
German	1.95	0.86	**Ref.**	< .001									< .001	< .001
Others	1.49	0.85	< .001	**Ref.**									< .001	< .001
Grade														
5th	1.51	0.86	**Ref.**	1	1	1	.737	.002	< .001	.001	< .001	1	< .001	.002
6th	1.4	0.81	1	**Ref.**	1	.727	.006	< .001	< .001	< .001	< .001	1	.001	.001
7th	1.46	0.88	1	1	**Ref.**	.988	.036	< .001	< .001	< .001	< .001	1	< .001	< .001
8th	1.62	0.81	1	.727	.988	**Ref**.	.370	< .001	< .001	< .001	< .001	1	< .001	< .001
9th	1.79	0.8	.737	.006	.036	.370	**Ref.**	.008	< .001	.008	< .001	1	< .001	< .001
10th	2.04	0.89	.002	< .001	< .001	< .001	.008	**Ref.**	1	1	.215	.243	< .001	< .001
11th	2.15	0.84	< .001	< .001	< .001	< .001	< .001	1	**Ref.**	1	1	.045	< .001	< .001
12th	2.11	0.81	.001	< .001	< .001	< .001	.008	1	1	**Ref.**	.999	.109	< .001	< .001

12/13th	2.25	0.81	< .001	< .001	< .001	< .001	< .001	.215	1	.999	**Ref.**	.005				< .001	< .001
Others	1.54	1.02	1	1	1	1	1	.243	0.45	.103	.005	**Ref.**				.200	.255
								School level									
Upper secondary	1.55	0.83	**Ref.**	< .001												< .001	< .001
Lower secondary	2.03	0.85	< .001	**Ref.**												< .001	< .001
								School type									
(N)MS	1.34	0.77	**Ref.**	< .001	< .001	.007	< .001	< .001	.033	1	< .001	.745	< .001	.003	.722	< .001	< .001
AHS (lower secondary)	1.95	0.78	< .001	**Ref.**	< .001	1	.207	.998	.971	.981	1	< .001	1	.144	.677	< .001	< .001
AHS (upper secondary)	2.31	0.80	< .001	< .001	**Ref.**	.303	1	< .001	.003	.652	< .001	< .001	.171	.890	.003	< .001	< .001
BMS	1.94	0.74	.007	1	.303	**Ref.**	.688	1	.999	.991	1	.223	1	.208	.939	.002	.039
BORG	2.28	0.88	< .001	.207	1	.688	**Ref.**	.033	.061	.718	.204	< .001	.665	.907	.023	< .001	.015
BHAK (economy)	1.87	0.86	< .001	.998	< .001	1	.033	**Ref.**	1	.996	.992	.010	1	.080	.944	< .001	< .001
BHAS	1.78	0.81	.033	.971	.003	.999	.061	1	**Ref.**	1	.950	.746	.989	.049	1	.002	.005
HTL	1.5	1.07	1	.981	.652	.991	.718	.996	1	**Ref.**	.977	1	.981	.386	1	.200	.587
HLW (agriculture)	1.96	0.84	< .001	1	< .001	1	.204	.992	.950	.977	**Ref.**	< .001	1	.150	.616	< .001	< .001
PTS	1.51	0.67	.745	< .001	< .001	.223	< .001	.010	.746	1	< .001	**Ref.**	.030	.006	1	< .001	.001
BAfEP	1.97	0.76	< .001	1	.171	1	.665	1	.989	.981	1	.030	**Ref.**	.212	.797	.011	.024
HBLA	2.61	0.55	.003	.144	.890	.208	.907	.080	.049	.386	.150	.006	.212	**Ref.**	.019	.200	.172
Others	1.64	0.86	.722	.677	.003	.939	.023	.944	1	1	.616	1	.797	.019.	**Ref.**	.034	.219

Total	1.87	0.87

Parameter	Factors of t-test / analyses of variance					
	Significance of Levene´s test	Number of degrees of freedom (df)	t-test / ANOVA (F / Welch-F)	p	d / η²	95 % CI of d / η²
			Analyses of variance			
Sex	.152	2, 1725	9.811	< .001	.011	.003 – .022
Grade	.467	9, 1718	20.278	< .001	.096	.067 – .118
School type	.046	12, 140.757	26.303	< .001	.151	.117 – .176
State (in combination with lower and upper secondary school)	.102	4, 1719	1.824	.122	.004	
State (in combination with lower and upper secondary school as well as school type)	.006	3, 1680	3.056	.027*	.005	
			t-tests			
First Language	.343	1726	8.243	< .001	0.531	0.404 – 0.659
Secondary school level (lower – upper)	.254	1703	10.885	< .001	0.567	0.463 – 0.671

* No significance because significance threshold was lowered to .01 due to inhomogeneity of variance

Summary:

There was no significant difference between male and female participants. Comparing lower and upper secondary students of same school types across the three participating states also showed no significant differences ($F(3, 1680) = 3.056$, $p = .027$, $\eta 2 = .005$; significance threshold was lowered to 0.01 due to inhomogeneity of variance (Levene's test $p = .006$) (Bühl, 2016)). However, students with German as first language yielded significantly better results. ANOVA also revealed a significant group effect comparing lower and upper secondary students. Additionally, significant differences were noted between grades. Most upper secondary grades (10 – 12/13) scored significantly better than all lower secondary grades. No significant difference was observed between grades 12/13 and grade 10. But significant differences could be found between school types. For example, students of lower secondary high school gained significantly more points than students from middle school.

Table S7. Students' choices concerning differences between bacteria and viruses. For "combination" a cut-off was set at 5 % because of the high number of combinations. "Single answers and in combination" comprises the sums of all participants who had ticked of the respective option either alone or in combination with other options.

	Which of the following statements concerning bacteria and viruses are correct?											
	Single answers (in %)					Combinations (in %)		Single answers and in combination (in %)				
Grade	Bacteria are smaller	Bacteria are more complex	Viruses are more dangerous	Antibiotics can be used against bacteria and viruses	Uncertain/ don't know	Bacteria more complex + viruses more dangerous	Bacteria smaller + viruses more dangerous	Bacteria smaller	Bacteria more complex	Viruses more dangerous	Antibiotics against bacteria and viruses	Uncertain/ don't know
8th	5.9	11.3	16.9	4.4	21.6	8.4	6.6	22.2	30.6	45.6	23.1	26.9
10th	4.0	24.3	14.3	3.7	16.5	9.6	4.0	18.8	46.3	37.9	18.0	22.4
12/13th	4.9	23.7	14.7	3.1	18.3	8.9	3.1	17.4	43.8	38.4	19.2	22.8
All grades	7.0	18.3	15.5	3.7	19.5	8.5	5.6	22.7	37.7	40.1	18.0	24.0

Table S8. Knowledge score for the domain virus of study A in relation to specific demographic parameters.

	Achieved points		Post-hoc-test (GT2 Hochberg or Games-Howell) / t-test				Test of normal distribution	
Parameter	M	SD	P				Kolmogorov-Smirnov (p)	Shapiro-Wilk (p)
				Sex				
Male	3.80	1.96	*Reference*	.060	< .001		< .001	< .001
Female	3.52	1.88	.060	*Reference*	< .001		< .001	< .001
Diverse	2.07	1.18	< .001	< .001	*Reference*		.200	.367
				Age				
below 21	2.83	1.42	*Reference*	< .001	< .001	< .001	< .001	< .001
21 to 40	4.76	2.13	< .001	*Reference*	.006	.396	< .001	< .001
41 to 60	4.14	1.80	< .001	.006	*Reference*	.985	.002	.001
over 60	4.25	2.11	< .001	.396	.985	*Reference*	.200	.757
				Level of education				
No final secondary degree	2.66	1.32	*Reference*	1	< .001	< .001	.021	< .001
GCSE	2.68	1.29	1	*Reference*	< .001	< .001	.001	< .001
A-levels	3.97	1.90	< .001	< .001	*Reference*	< .001	< .001	< .001
University degree	4.95	2.01	< .001	< .001	< .001	*Reference*	.001	< .001
				First Language				
German	3.72	1.93	*Reference*	< .001			< .001	< .001
Others	2.70	1.55	< .001	*Reference*			< .001	< .001
				Prior knowledge about viruses				
Yes	5.62	2.05	*Reference*	< .001			< .001	< .001
No	3.21	1.62	< .001	*Reference*			< .001	< .001
				Interest in viruses				
Yes	4.05	2.03	*Reference*	< .001			< .001	< .001
No	3.25	1.74	< .001	*Reference*			< .001	< .001
Total	3.61	1.92						

	Factors of t-test / analyses of variance

Parameter	Significance of Levene´s test	Number of degrees of freedom (df)	t-test / ANOVA (F / Welch-F)	p	d / η^2	95 % CI of d / η^2
			Analyses of variance			
Sex	.042	2, 54.616	18.932	< .001	.018	.005 – .036
Age	< .001	3, 191.792	75.444	< .001	.195	.153 – .235
Level of education	< .001	6, 162.366	49.694	< .001	.235	.189 – .274
			t-tests			
First Language	< .001	156.656	6.360	< .001	0.536	0.338 – 0.733
Prior knowledge about viruses	< .001	211.252	14.418	< .001	1.419	1.243 – 1.595
Interest in viruses	< .001	909.690	6.702	< .001	0.427	0.303 – 0.551

Summary:

There was no significant difference between males and females. The age group 21 – 40 performed best. The knowledge difference was greatest between those below 21 and those between 21 and 40, yet it was also significant between those being between 21 and 40 and those being between 41 and 60. With respect to education, those with a university degree performed significantly better than all other groups. Participants with A-levels also reached significantly more points than those with GCSE and without final secondary degree. Furthermore, people who regarded themselves as equipped with some virology knowledge performed significantly better than those who did not. Participants with German as mother tongue reached significantly more points than those with other languages.

Table S9. Knowledge score for the domain virus of study B in relation to specific demographic parameters.

	Achieved points		Result of post-hoc-test (GT2 Hochberg or Games-Howell) / t-test										Test of normal distribution	
Parameter	M	SD	p										Kolmogorov-Smirnov (p)	Shapiro-Wilk (p)
						Sex								
Male	2.55	1.48	**Ref.**	.094	.001								< .001	< .001
Female	2.4	1.34	.094	**Ref.**	.011								< .001	< .001
Diverse	1.86	1.26	.001	.011	**Ref.**								.004	< .001
						First Language								
German	2.51	1.42	**Ref.**	< .001									< .001	< .001
Others	2.07	1.21	< .001	**Ref.**									< .001	< .001
						Grade								
5th	2.11	1.47	**Ref.**	.954	1	.999	.917	.339	.080	.314	.021	1	.025	< .001
6th	1.8	1.14	.954	**Ref.**	.996	.980	.001	< .001	< .001	< .001	< .001	.998	.171	.025
7th	1.96	1.23	1	.996	**Ref.**	1	.068	.001	< .001	.004	< .001	1	.001	< .001
8th	1.95	1.02	.999	.980.	1	**Ref.**	< .001	< .001	< .001	< .001	< .001	1	< .001	< .001
9th	2.42	1.38	.917	001	.068	< .001	**Ref.**	.555	.036	.669	.003	.978	< .001	< .001
10th	2.65	1.38	.339	< .001	.001	< .001	.555	**Ref.**	.949	1	.476	.726	< .001	< .001
11th	2.82	1.32	.080	< .001	< .001	< .001	.036	.949	**Ref.**	1	.997	.425	< .001	< .001
12th	2.73	1.58	.314	< .001	.004	< .001	.669	1	1	**Ref.**	.965	.656	.017	< .001

12/13th	2.95	1.61	.021	< .001	< .001	< .001	.003	.476	.997	.965	**Ref.**	.241				< .001	< .001
Others	2.06	1.52	1	.998	1	1	.978	.726	.425	.656	.241	**Ref.**				.048	.005
									School level								
Upper secondary	1.95	1.12	**Ref.**	< .001												< .001	< .001
Lower secondary	2.68	1.45	< .001	**Ref.**												< .001	< .001
									School type								
(N)MS	1.70	0.96	**Ref.**	< .001	< .001	.235	< .001	< .001	.048	.999	< .001	.938	< .001	.321	.042	< .001	< .001
AHS (lower secondary)	2.41	1.3	< .001	**Ref.**	< .001	1	.166	1	1	1	1	.010	.747	1	1	< .001	< .001
AHS (upper secondary)	3.24	1.61	< .001	< .001	**Ref.**	.419	.961	< .001	.001	.961	< .001	< .001	.267	.414	.094	< .001	< .001
BMS	2.51	1.56	.235	1	.419	**Ref.**	.971	1	1	1	1	.648	1	1	1	.003	< .001
BORG	2.97	1.43	< .001	.166	.961	.971	**Ref.**	.224	.345	.997	.089	< .001	1	.942	.868	.200	.003
BHAK (economy)	2.44	1.31	< .001	1	< .001	1	.224	**Ref.**	1	1	1	.003	.834	1	1	< .001	< .001
BHAS	2.34	1.27	.048	1	.001	1	.345	1	**Ref.**	1	1	.541	.847	1	1	.058	.027
HTL	2.3	2.0	.999	1	.961	1	.997	1	1	**Ref.**	1	1	1	1	1	.089	.124
HLW (agriculture)	2.39	1.33	< .001	1	< .001	1	.089	1	1	1	**Ref.**	.006	.585	1	1	< .001	< .001
PTS	1.88	0.92	.938	.010	< .001	.648	< .001	.003	.541	1	.006	**Ref.**	< .001	.657	.341	.200	.399
BAfEP	2.76	1.09	< .001	.747	.267	1	1	.834	.847	1	.585	< .001	**Ref.**	.999	.998	.200	.908
HBLA	2.49	9.82	.321	1	.414	1	.942	1	1	1	1	.657	.999	**Ref.**	1	.200	.363
Others	2,49	1.30	.042	1	.094	1	.868	1	1	1	1	.341	.998	1	**Ref.**	.044	.159

Total	2.44	1.4

	Factors of t-test / analyses of variance					
Parameter	Significance of Levene´s test	Number of degrees of freedom (df)	t-test / ANOVA (F / Welch-F)	p	d / η^2	95 % CI of d / η^2
			Analyses of variance			
Sex	.006	2, 140.165	7.639	.001	.008	.001 – .018
Grade	< .001	9, 307.606	16.901	< .001	.073	0.47 – 0.93
School type	< .001	12, 140.527	23.453	< .001	.135	.101 – .159
State (in combination with lower and upper secondary school)	< .001	4, 1719	1.025	.393	.002	
State (in combination with lower and upper secondary school as well as school type)	< .001	3, 1680	1.001	.391	.002	
			t-tests			
First Language	.002	461.942	5.479	< .001	0.318	0.191 – 0.455
Secondary school level (lower – upper)	< .001	1324.697	11.360	< .001	0.539	0.436 – 0.643

Summary:

There was no significant difference between male and female students. Again, there were no significant differences between the three states when comparing lower and upper secondary students of the same school types. Students‘ first language significantly influenced achievement. Those with German as first language gained in average 0.44 points more than those with other first languages. Students from upper secondary gained significantly more points than those from lower secondary. Grade also had a significant effect. Grade-12/13 students and grade-10 students scored significantly better than students of grade 8. Within lower secondary, achievement did not differ significantly between grades. There were no significant differences between grade 12/13 and grade 10 students. Yet school type had a significant effect.
